# Human iPSC-based models highlight defective glial and neuronal differentiation from neural progenitor cells in metachromatic leukodystrophy

**DOI:** 10.1038/s41419-018-0737-0

**Published:** 2018-06-13

**Authors:** Giacomo Frati, Marco Luciani, Vasco Meneghini, Silvia De Cicco, Marcus Ståhlman, Maria Blomqvist, Serena Grossi, Mirella Filocamo, Francesco Morena, Andrea Menegon, Sabata Martino, Angela Gritti

**Affiliations:** 1 San Raffaele Telethon Institute for Gene Therapy (SR-Tiget), IRCCS San Raffaele Scientific Institute, Via Olgettina 58, 20132 Milano, Italy; 2 Department of Molecular and Clinical Medicine, University of Gothenburg and Sahlgrenska University Hospital, SE-41101 Gothenburg, Sweden; 3 Department of Clinical Chemistry and Transfusion Medicine, Institute of Biomedicine, Sahlgrenska Academy, University of Gothenburg, SE-41101 Gothenburg, Sweden; 4 UOSD Centro di diagnostica genetica e biochimica delle malattie metaboliche, IRCCS G. Gaslini Institute, Via G. Gaslini, 16147 Genova, Italy; 5 Department of Chemistry, Biology, and Biotechnologies, University of Perugia, Via del Giochetto, 06126 Perugia, Italy; 6 Experimental Imaging Center, IRCCS San Raffaele Scientific Institute, Milan, Italy; 7 Present Address: Institute Imagine, 24 Boulevard du Montparnasse, 75015 Paris, France; 8 Present Address: Deutsches Zentrum für Neurodegenerative Erkrankungen, Otfried-Müller Str.23, 72076 Tübingen, Germany

## Abstract

The pathological cascade leading from primary storage to neural cell dysfunction and death in metachromatic leukodystrophy (MLD) has been poorly elucidated in human-derived neural cell systems. In the present study, we have modeled the progression of pathological events during the differentiation of patient-specific iPSCs to neuroepithelial progenitor cells (iPSC-NPCs) and mature neurons, astrocytes, and oligodendrocytes at the morphological, molecular, and biochemical level. We showed significant sulfatide accumulation and altered sulfatide composition during the differentiation of MLD iPSC-NPCs into neuronal and glial cells. Changes in sulfatide levels and composition were accompanied by the expansion of the lysosomal compartment, oxidative stress, and apoptosis. The neuronal and glial differentiation capacity of MLD iPSC-NPCs was significantly impaired. We showed delayed appearance and/or reduced levels of oligodendroglial and astroglial markers as well as reduced number of neurons and disorganized neuronal network. Restoration of a functional Arylsulfatase A (ARSA) enzyme in MLD cells using lentiviral-mediated gene transfer normalized sulfatide levels and composition, globally rescuing the pathological phenotype. Our study points to MLD iPSC-derived neural progeny as a useful in vitro model to assess the impact of ARSA deficiency along NPC differentiation into neurons and glial cells. In addition, iPSC-derived neural cultures allowed testing the impact of ARSA reconstitution/overexpression on disease correction and, importantly, on the biology and functional features of human NPCs, with important therapeutic implications.

## Introduction

Metachromatic Leukodystrophy (MLD) is a rare genetic lysosomal storage disorder (LSD) caused by the functional deficiency of Arylsulfatase A (ARSA; EC 3.1.6.8). ARSA catalyzes the desulfation of 3-O-sulfogalactosylceramide (sulfatide)^1^, a sphingolipid that plays key roles in the development and function of myelin-forming cells as well as in the organization and maintenance of myelin structure^2–5^. Sulfatide storage mainly affects oligodendrocytes and Schwann cells leading to progressive demyelination and dysfunction of the central and peripheral nervous system (CNS, PNS). Rapid motor and cognitive decline, and premature death are typical of the late-infantile forms of MLD. Juvenile forms display a slower progression of motor symptoms often preceded by cognitive and behavioral problems, which characterize the adult form^6–8^.

Sulfatide occurs as a collection of cell- and tissue-specific molecular species that differ in the length of the acyl chain, the presence of saturated or unsaturated bonds and hydroxylation^1,2^.

In the nervous tissue, sulfatide species containing long-chain fatty acids (≥C24) are abundant in myelin. Short-chain fatty acid sulfatide species (C16−C18) are present in the cortical gray matter^9^ and in immature oligodendrocytes^10^, astrocytes, and neurons^11–13^. Preclinical studies in galactolipid-deficient and ARSA-deficient mice and neural cells^6,14,15^, as well as clinical observations^16^, suggest that sulfatide storage affects early oligodendroglial development and that sulfatide load beyond myelin might contribute to MLD pathology. Sulfatide is involved in oligodendrocyte survival and proliferation^17–20^, negatively regulates oligodendrocyte terminal differentiation^17^, delays myelin formation^21^ and has been proposed as a novel myelin-associated inhibitor of axon regeneration in CNS neurons^22^. Accumulation of sulfatide alters neuronal cell morphology and organization leading to axonal degeneration^23,24^ and possibly contributing to motor dysfunctions in MLD patients^16^. Finally, an excess of sulfatide has been associated with gray matter astrogliosis^25,26^. Overall, the molecular events that link the primary storage to the appearance and progression of cell-type-specific pathological phenotypes are poorly elucidated. Also, it remains unknown whether the observations made in murine systems recapitulate critical aspects of the human disease.

The derivation of patient-specific induced pluripotent stem cells (iPSCs) and their differentiation in cell types of interest have emerged as a powerful tool for human disease modeling and therapeutic screening^27–30^. The establishment of MLD patient-specific iPSCs, the engineering of these cells to overexpress a functional human *ARSA* gene and their efficient differentiation in neural progenitors with therapeutic potential have been recently reported^31,32^. However, these studies were not focused on disease modeling and did not investigate the mechanisms of disease in MLD iPSCs and neural progeny.

In the present study, we took advantage of MLD iPSCs to model the impact of ARSA deficiency on sulfatide storage and secondary pathogenic events through the differentiation of iPSCs to neuroepithelial progenitor cells (iPSC-NPCs), neurons, astrocytes, and oligodendrocytes. We also highlighted the impact of ARSA reconstitution/overexpression (achieved by lentiviral-mediated gene transfer) on disease correction as well as on the biology of human NPCs and neuronal/glial progeny.

## Results

The normal donor (ND1, ND2) and patient-specific (MLD1, MLD2) iPSC lines/clones used here have been previously described^32^. MLD iPSC clones expressing supraphysiological ARSA activity (MLD-ARSA) were obtained by lentiviral (LV)-mediated gene transfer of a functional human *ARSA* gene^32^. The iPSC clones used are listed in Supplementary Methods.

### Moderate lysosomal expansion and oxidative stress in MLD iPSCs

**Fig. 1:**
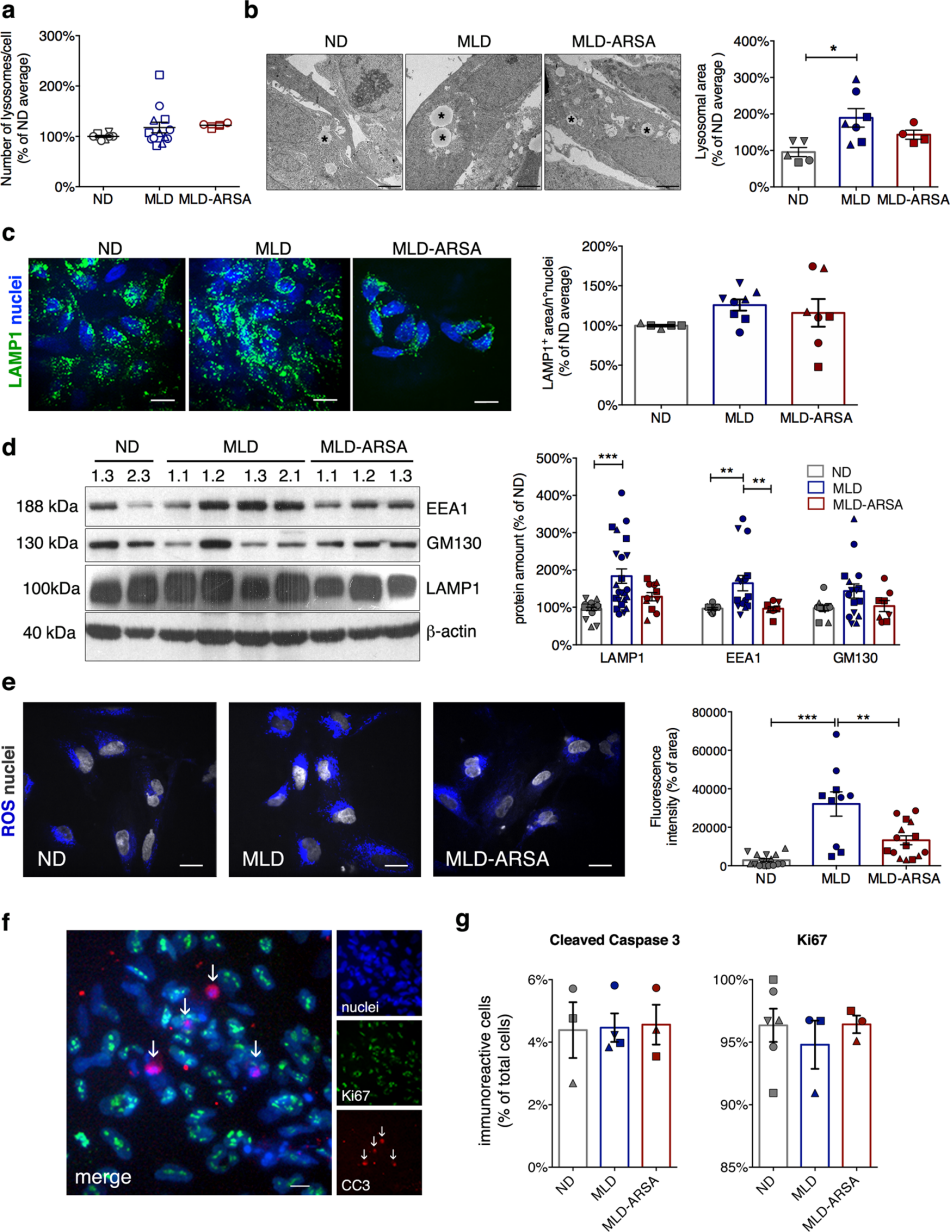
Moderate lysosomal expansion and oxidative stress in MLD iPSCs. **a** Dot-plot showing the number of lysosomes/cell in ND, MLD, and MLD-ARSA iPSC clones. *n* = 6 independent experiments, 2–4 clones/group with 2–4 replicates/clone. Clones used: ND 1.1 (O); ND 1.3 (□); ND 2.2 (Δ); ND 2.3 (∇); MLD 1.1 (O); MLD 1.2 (□); MLD 1.3 (Δ); MLD-ARSA 1.1 (O); MLD-ARSA 1.2 (□). Data were normalized to the average ND value of each experiment. Data were analyzed by one-Way ANOVA followed by Bonferroni’s multiple comparisons test. **b** Representative transmission electron microscopy (EM) images of ND, MLD, and MLD-ARSA iPSCs and quantification of the lysosomal area performed on EM images. Asterisks identify lysosomal vesicles. Scale bars: 2 µm. We counted 60–70 lysosomes/clone; 2–4 clones/group with 1–3 replicates/clone. Clones used: ND 1.1 (O); ND 1.3 (□); ND 2.2 (Δ); ND 2.3 (∇); MLD 1.1 (O); MLD 1.2 (□); MLD 1.3 (Δ); MLD-ARSA 1.1 (O); MLD-ARSA 1.2 (□). Bars indicate the mean ± SEM. Data were normalized to the average ND value of each experiment. Data were analyzed by one-way ANOVA followed by Bonferroni’s multiple comparisons test. *p < 0.05. **c** Representative immunofluorescence pictures and quantification of the LAMP1-immunopositive area (green) in ND, MLD, and MLD-ARSA iPSCs. Nuclei are counterstained with To-Pro III (blue). Scale bar: 20 µm. Data are expressed as px^2^/number of cells with a defined nucleus and are normalized to the average ND value of each experiment. Bars indicate the mean ± SEM. *n* = 5 experiments, 2–3 clones/group with 1–3 replicates/clone. Clones used: ND 1.3 (□); ND 2.1(Δ); MLD 1.1 (O); MLD 1.2 (□); MLD 1.3 (Δ); MLD2.1 (∇); MLD-ARSA 1.1 (O); MLD-ARSA 1.2 (□); MLD-ARSA 1.3 (Δ). **d** Western blot showing the expression of early endosomal antigen protein 1 (EEA1), GM130 (Golgi marker) and LAMP1 in representative ND, MLD, and MLD-ARSA iPSC clones. β-actin was used as loading control (10 µg of total proteins per lane). The bar graph shows the quantification of WB bands. Data are normalized on β-actin and expressed as the percentage of ND in each experiment; *n* = 3–8 independent experiments, 4 clones/group with 1–6 replicates/clone. Clones used: ND 1.1 (O); ND 1.3 (□); ND 2.2 (Δ); ND 2.3 (∇); MLD 1.1 (O); MLD 1.2 (□); MLD 1.3 (Δ); MLD 2.1 (∇); MLD-ARSA 1.1 (O), MLD-ARSA 1.2 (□); MLD-ARSA 1.3 (Δ); MLD-ARSA 2.1(∇). Data were analyzed by one-way ANOVA followed by Bonferroni’s multiple comparisons test. **p < 0.01, ***p < 0.001. **e** Representative confocal fluorescence pictures and quantification of fluorescence intensity of ND, MLD, and MLD-ARSA iPSCs stained with Deep Red CellROX^®^ to detect reactive oxygen species (ROS; blue). Nuclei counterstained with Hoechst (white). Scale bar: 10 µm. We analyzed *n* = 10 fields/coverslip/clone in five independent experiments; 2–3 clones/group with 3–6 replicates/clone. Clones used: ND 1.1 (O); ND 2.2 (Δ); ND 2.3 (∇); MLD 1.1 (O); MLD 1.2 (□); MLD-ARSA 1.1 (O), MLD-ARSA 1.2 (□); MLD-ARSA 1.3 (Δ). Data were analyzed by one-way ANOVA followed by Bonferroni’s multiple comparisons test. **p < 0.01; ***p < 0.001. **f** Representative immunofluorescence picture of ND iPSCs expressing Ki67 (green) and cleaved caspase-3 (CC3, red; arrows). Nuclei are counterstained with DAPI (blue). Single channels and the merged image are shown. Scale bar: 10 µm. **g** Graphs showing percentages of cleaved caspase-3- and Ki67-positive cells (on total nuclei) in ND, MLD, and MLD-ARSA iPSCs. Data are expressed as the mean ± SEM; *n* = 3 independent experiments, 3–4 clones/group with 1–2 replicates/clone. Clones used: ND 1.1 (O); ND 1.3 (□); ND 2.2 (Δ); MLD 1.1 (O); MLD 1.2 (□); MLD 1.3 (Δ); MLD 2.1 (∇); MLD-ARSA 1.1 (O), MLD-ARSA 1.2 (□); MLD-ARSA 1.3 (Δ)

Lysotracker assay showed a comparable number of lysosomes/cell in ND, MLD, and MLD-ARSA cultures (Fig. 1a). Electron microscopy (EM) analysis revealed increased lysosomal size in MLD as compared to ND iPSCs, which was partially rescued in MLD-ARSA iPSCs (Fig. 1b). Lysosomal expansion in MLD iPSCs was further suggested by increased expression of the lysosomal-associated membrane protein 1 (LAMP1) (Fig. 1c, d). Increased expression of the *cis*-Golgi matrix protein GM130 and of the early endosome antigen 1 (EEA1) protein (Fig. 1d) in MLD iPSCs may link lysosomal expansion to impaired endo-lysosomal trafficking^33^ and consequent production of reactive oxygen species (ROS)^34^ (Fig. 1e). Endo-lysosomal defects and oxidative stress were ameliorated or counteracted in MLD-ARSA cells (Fig. 1d, e). The low percentages of apoptotic cells in MLD iPSCs (Fig. 1f, g) suggest that increased ROS levels are not sufficient to shift the balance between the mitogen-dependent cell proliferation (>90% of Ki67^+^ cells; Fig. 1f, g) and cell death.

Overall these results suggest that ARSA deficiency is responsible for the moderate expansion of the endolysosome system and intracellular ROS production in MLD iPSCs without major impact on cell survival and proliferation.

### The lysosomal expansion, ROS production, and apoptosis are exacerbated in MLD iPSC-derived NPCs and neuronal/glial progeny

**Fig. 2:**
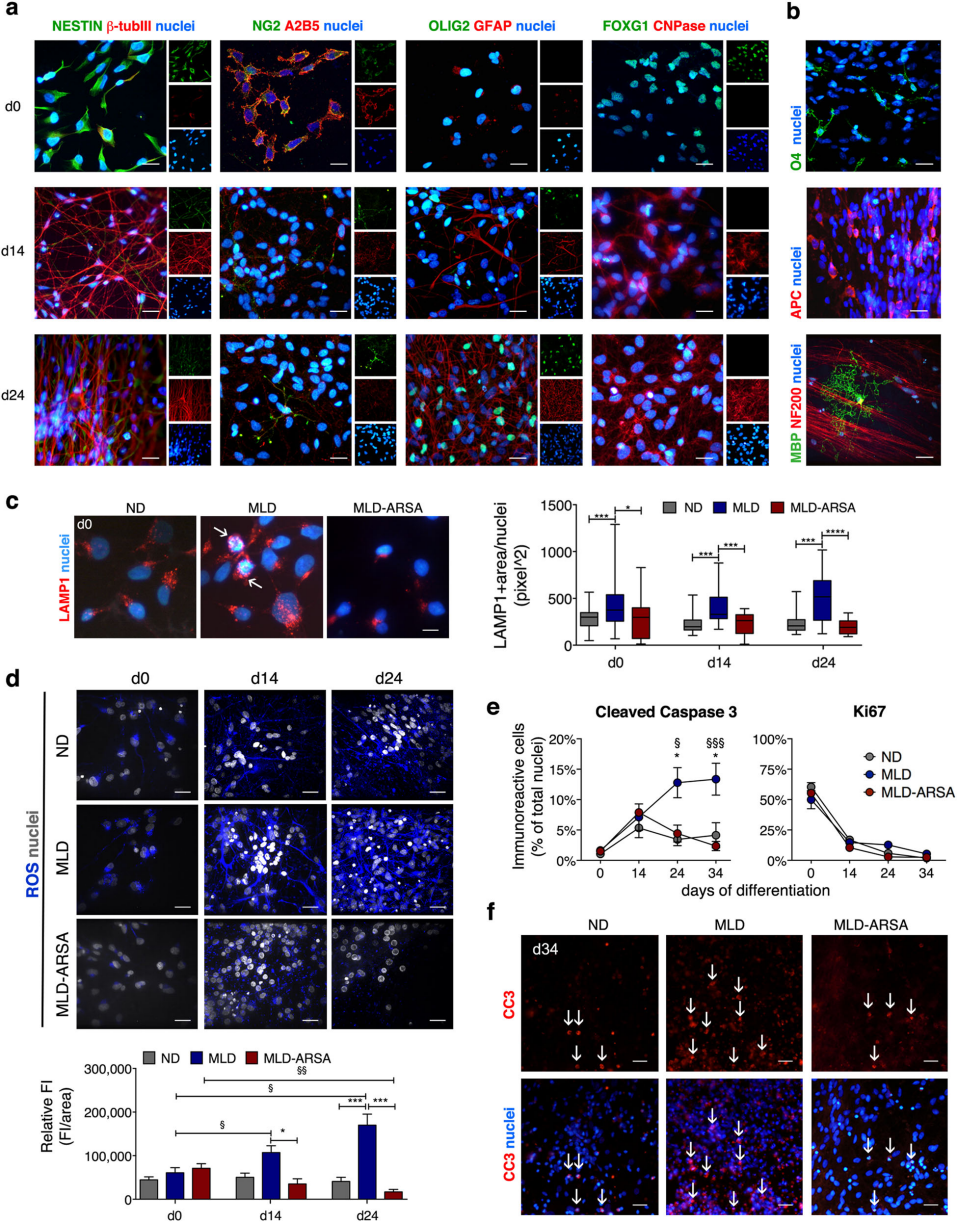
Lysosomal expansion, ROS production, and apoptosis in MLD iPSC-derived NPCs and neural progeny. **a** Representative immunofluorescence pictures (single channels and merged images) showing the expression of NPC markers (NESTIN, FOXG1), oligodendroglial cell markers (A2B5, NG2, OLIG2, CNPase), astroglial (GFAP), and neuronal (β-tubulin III) markers at different time points of differentiation (d0, d14, and d24; clone ND 1.3). Lineage markers are shown in red or green. Nuclei are counterstained with DAPI (blue). Scale bars: 20 µm. **b** Representative immunofluorescence merged pictures of ND cultures (clone ND 1.3) showing cells expressing oligodendroglial markers (O4, APC; d24 of differentiation) and one MBP-positive cell (green) interacting with neurons (NF200, red; d34 of differentiation). Nuclei counterstained with DAPI (blue). Scale bars: 20 µm. **c** Representative immunofluorescence pictures (d0) and quantification (d0, d14, d24) of the immunopositive area showing increased LAMP1 expression (red) in MLD cells as compared to ND cells, and rescue to normal levels in MLD-ARSA cultures. Nuclei are counterstained with DAPI (blue). Arrows point to enlarged lysosomal vesicles. Scale bar: 10 µm. Data in the graph are the mean ± SEM; *n* = 4–6 independent experiments, 3 clones/group with 2 replicates/clone. Clones used: ND 1.1; ND 1.3; ND 2.2; MLD 1.1 MLD 1.3; MLD 2.1; MLD-ARSA 1.1; MLD-ARSA 1.2; MLD-ARSA 1.3. Data relative to each time point were analyzed by one-way ANOVA followed by Bonferroni’s multiple comparison tests; *p < 0.05; ***p < 0.001; ****p < 0.0001. **d** Representative confocal fluorescence pictures and quantification of the relative fluorescence intensity (FI) of ND, MLD, and MLD-ARSA iPSC-derived NPCs and neural progeny at different days of differentiation (d0, d14, d24) stained with Deep Red CellROX^®^ to detect reactive oxygen species (ROS). Nuclei are counterstained with Hoechst (white). Scale bar: 20 µm. Data in the graph are the mean ± SEM. *n* = 10 fields/coverslip/clone in 4–6 independent experiments, 3 clones/group with 2 replicates/clone. Clones used: ND 1.1, ND 1.3, ND 2.3, MLD 1.1, MLD 1.3, MLD 2.1, MLD-ARSA 1.1, MLD-ARSA 1.2, MLD-ARSA 1.3. Data relative to each time point were analyzed by one-way ANOVA followed by Bonferroni’s multiple comparison tests; *p < 0.05; ***p < 0.001; §*p* < 0.05; §§*p* < 0.01. **e** Percentage of cleaved caspase-3- and Ki67-positive cells in ND, MLD, and MLD-ARSA cultures at different days of differentiation (d0, d14, d24, d34). Data are expressed as the mean ± SEM; *n* = 4–6 independent experiments, 2–3 clones/group in duplicate. Clones used: ND 1.1, ND 1.3, ND 2.3; MLD 1.1, MLD 1.3, MLD 2.1; MLD-ARSA 1.1, MLD-ARSA 1.3. Data analyzed with two-way ANOVA followed by Bonferroni’s multiple comparisons test. *p < 0.05 MLD vs. ND; §*p* < 0.05 MLD vs. MLD-ARSA; §§§*p* < 0.001 MLD vs. MLD-ARSA. **f** Representative immunofluorescence pictures showing cleaved caspase-3-positive cells (CC3, red) in ND, MLD, and MLD-ARSA cultures at d34 of differentiation. Nuclei are counterstained with DAPI (blue). Arrows identify cells in the red channel and in merged pictures. Scale bar, 50 µm

We next sought to assess the impact of ARSA deficiency on the endo-lysosomal system and oxidative stress during the pluripotent to neural transition. We differentiated iPSCs into neuroepithelial progenitor cells (NPCs) using a published protocol^35,36^ (Supplementary Figure 1a−e). NPC populations could be generated and expanded with similar efficiency from ND, MLD and MLD-ARSA iPSCs (Supplementary Figure 1b−c, e−f). The rapid downregulation of PAX6 expression in iPSC-NPCs (Supplementary Figure 1f) suggested their predisposition to rapid lineage commitment/differentiation^37,38^. We adopted a protocol known to produce mixed populations of neurons, astrocytes, and oligodendrocytes (the cell type that is most affected in MLD) at different stages of differentiation/maturation^32^. NPCs were exposed to increasing concentrations of growth factors (GFs) promoting the differentiation of glial progenitors (Glial Differentiation Medium, GDM; Supplementary Figure 1a)^39^. 10 days after the switch to GDM we observed a substantial reduction of NESTIN^+^ and NG2^+^ precursors, and the appearance/increase of A2B5^+^ glial progenitors (≈20%), oligodendroglial progenitors/immature oligodendrocytes (Olig2^+^ and O4^+^ cells; ≈10% and ≈3%, respectively) and mature oligodendrocytes (APC^+^ and CNPase^+^ cells; ≈10% and ≈20%, respectively). Furthermore, ≈5% and ≈10% of cells in these cultures expressed astroglial (GFAP) and neuronal (β-Tubulin III) markers, respectively (Fig. 2a and Supplementary Table 1; d14). Exposure of cultures to a GF-deprived medium (Glial Maturation Medium; GMM) for additional 10 days (Supplementary Figure 1a) promoted oligodendroglial, astroglial, and neuronal differentiation, as demonstrated by further reduction of A2B5^+^ and NG2^+^ cells and significant increment of cells expressing APC, GFAP, and β-Tubulin III (Fig. 2a and Supplementary Table 1; d24). At d24, an electrophysiological analysis (MEA assay) showed a minimal spontaneous (TTX-sensitive) neuronal activity in ND, MLD, and MLD-ARSA cultures (Supplementary Figure 2). Also, we did not detect cells expressing myelin basic protein (MBP), which appeared later in the cultures and never exceeded the 2% of the total cells (Fig. 2b). Overall these data indicate that the protocol applied here drives the effective differentiation of iPSC-NPCs toward neuronal, astroglial, and oligodendroglial progeny (as also indicated by the progressive increase of ARSA activity in ND and MLD-ARSA cells; Supplementary Figure 3a) but supports limited functional maturation.

MLD iPSC-NPCs displayed enlarged lysosomal compartment that was normalized in ARSA-overexpressing cells (Fig. 2c; d0). ROS and apoptotic cells were barely detectable in NPCs (Fig. 2d, e; d0). Mitogen withdrawal resulted in a progressive decrease of Ki67^+^ proliferating cells in all the cell populations (Fig. 2e**)**. However, only MLD cultures showed an exacerbated ROS production (Fig. 2d) that was paralleled by an increased apoptosis (d24 and d34; Fig. 2e, f). Both pathological hallmarks were counteracted in MLD-ARSA cells (Fig. 2d−f).

### Atypical neuronal and glial differentiation of MLD NPCs is rescued by enzymatic correction: impact of ARSA overexpression

We next investigated the impact of the disease-associated cell dysfunction (and the extent of recovery upon ARSA overexpression) in iPSC-NPC–derived astroglial, oligodendroglial, and neuronal progeny. To this end, we integrated IF and WB analysis evaluating the expression of markers of NPCs (NESTIN), glial progenitors (A2B5, NG2), oligodendroglial precursors (PDGFRα), and mature (non-myelinating) oligodendrocytes (Olig2, O4, CNPase and APC), astrocytes (GFAP) and neurons (β-Tubulin III, synaptotagmin 1).

**Fig. 3:**
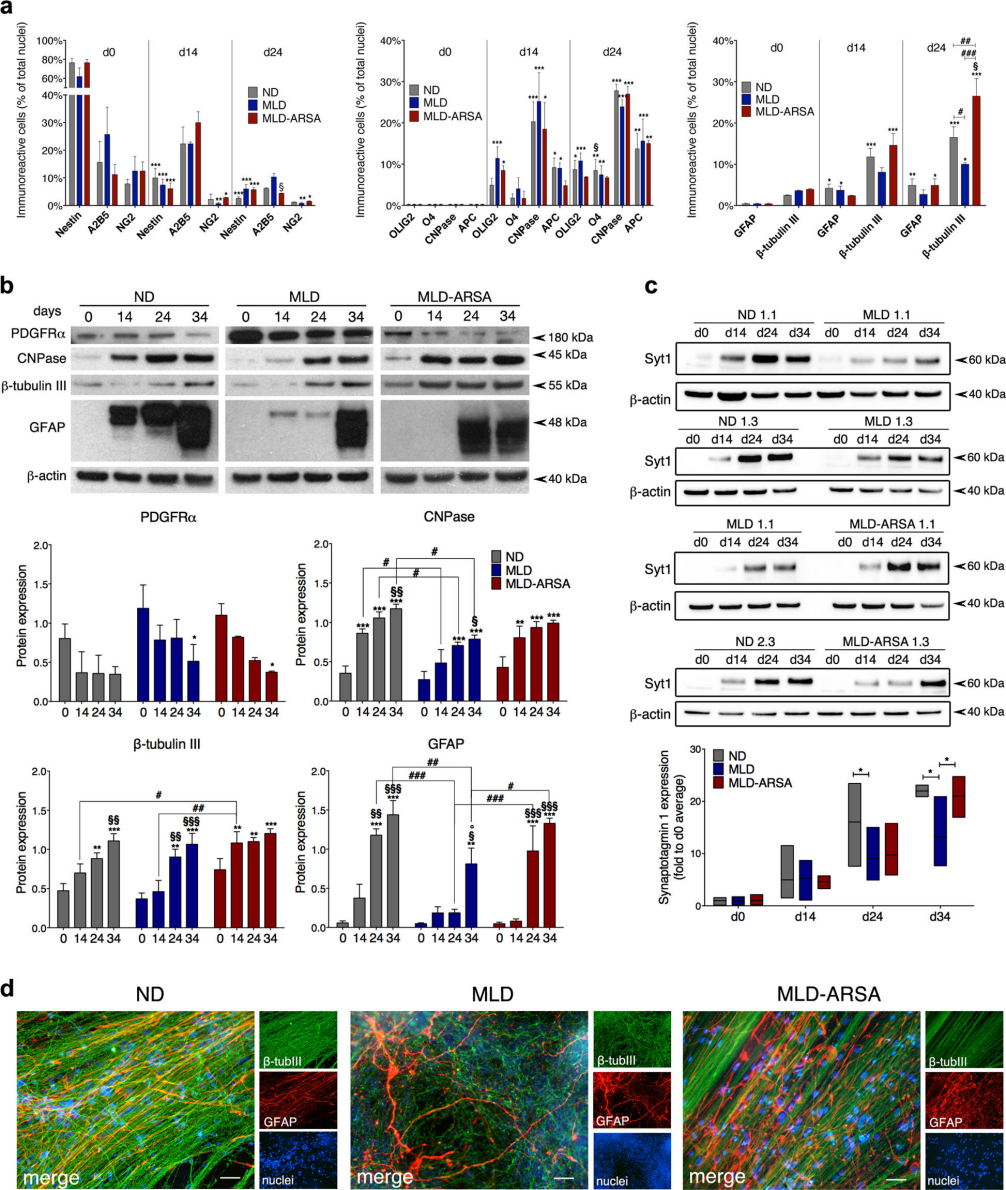
Impaired glial and neuronal differentiation of MLD iPSC-derived neural progeny. **a** Histogram plots showing the percentages of cells expressing markers of NPCs (NESTIN) and glial precursors (A2B5, NG2), oligodendrocytes (OLIG2, O4, CNPase, APC), astroglial (GFAP), and neuronal cells (β-tubulin III) at d0, d14, and d24 of differentiation in ND, MLD, and MLD-ARSA cultures. Data are expressed as the mean ± SEM; *n* = 6 (ND, MLD) and *n* = 4 (MLD-ARSA) independent experiments, 3 clones/group. Data were analyzed with two-way ANOVA followed by Tukey’s multiple comparison tests. *p < 0.05; **p < 0.01; ***p < 0.001 vs. d0 of the correspondent group (ND, MLD, and MLD-ARSA) for each marker; §*p* < 0.05 vs. d14 of the correspondent group (ND, MLD, and MLD-ARSA) for each marker. #*p* < 0.05; ##*p* < 0.01; ###*p* < 0.001. **b** Representative WB showing the expression of PDGFRα, CNPase, β-tubulin III, and GFAP proteins in ND 1.3-, MLD 2.1- and MLD-ARSA 1.2-derived cultures at different time points during the differentiation (d0, d14, d24, d34). β-actin was used as loading control (10 µg of total protein per lane). The bar graphs show the quantification of WB bands. Data are expressed as the fold to β-actin and represent the mean ± SEM; *n* = 3–6 experiments, 2 clones/group. Data were analyzed by two-way ANOVA followed by Tukey’s multiple comparison tests. *p < 0.05; **p < 0.01; ***p < 0.001 vs. d0 of the correspondent group (ND, MLD, and MLD-ARSA). §*p* < 0.05; §§*p* < 0.01; §§§*p* < 0.001 vs. d14 of the correspondent group (ND, MLD, and MLD-ARSA). °*p* < 0.05 vs. ND and MLD-ARSA at d34; #*p* < 0.05; ##*p* < 0.01; ###*p* < 0.001. **c** Western blots showing the expression of synaptotagmin 1 (Syt1) in ND, MLD, and MLD-ARSA cultures at d0, d14, d24, and d34 of differentiation. Four blots were run in order to analyze *n* = 2−3 samples/group. Clones used: ND 1.1, ND 1.3, ND 2.3, MLD 1.1 MLD 1.3, MLD-ARSA 1.1, MLD-ARSA 1.3. Floating bars graph show the quantification of Syt1 after normalization on β-actin. Data are expressed as the fold on the d0 average value, which was similar in all samples. Data are expressed as the mean ± SEM; Data were analyzed by two-way ANOVA followed by Tukey’s multiple comparison tests. *p < 0.05. **d** Representative confocal pictures (single channels and merged images) showing neurons (β-tubulin III, green) and astrocyte (GFAP, red) in ND 2.3-, MLD 2.1- and MLD-ARSA 1.1-derived cultures at d34 of differentiation. Scale bars: 20 µm

Analysis of the cell type composition along the differentiation showed moderate but consistent differences in the oligodendroglial and astroglial cell populations in MLD as compared to ND and MLD-ARSA cultures (i.e. a slight increase of OLIG2^+^ cells at d14; a decrease of GFAP^+^ cells and CNPase^+^ cells at d24; Fig. 3a). The stable decrease of β-tubulin III^+^ cells in MLD as compared to ND cultures (significant difference at d24) suggested the occurrence of cell loss and/or delayed/reduced neuronal differentiation. This phenotype was rescued in MLD-ARSA cultures, which also showed higher percentages of β-tubulin III^+^ as compared to ND counterparts (d24), suggesting a boost of neuronal survival and/or differentiation due to ARSA overexpression (Fig. 3a and Supplementary Table 1).

The neuronal and glial defects highlighted by IF analysis were confirmed and extended by WB analysis (Fig. 3b). MLD cultures displayed reduced/delayed GFAP expression as compared to ND cultures (Fig. 3b). The occurrence of delayed oligodendroglial differentiation was indicated by the reduced CNPase expression and concomitant persistent expression of PDGFRα (a marker of oligodendroglial progenitors; up to d24), which was instead rapidly downregulated (already at d14) in ND cultures (Fig. 3b). PDGFRα downregulation was appreciable in MLD-ARSA cells with a different kinetics with respect to ND cells, suggesting that other factors besides ARSA deficiency might delay oligodendroglial differentiation. The delayed expression of β-tubulin III (compare ND and MLD at d0-d14; Fig. 3a, b) and the reduced expression of synaptotagmin 1 (a protein involved in neurotransmission) at d24 and d34 (Fig. 3c) further suggested impaired differentiation of MLD neurons. This phenotype was exacerbated during the differentiation, in line with the progressive cell dysfunction due to ARSA deficiency. ARSA overexpression largely rescued this defective phenotype **(**Fig. 3a−c**)**. The anticipated/increased expression of β-tubulin III protein in MLD-ARSA cultures as compared to ND counterparts is in line with IF data and suggests an impact of ARSA overexpression on the iPSC-NPC differentiation program.

The atypical neuronal and astroglial cell morphology in MLD as compared to ND cultures further indicated a defective differentiation program consequent to ARSA deficiency (Fig. 3d and Supplementary Figure 3b). MLD neurons were loosely arranged in networks as compared to the thick neuronal bundles observed in physiological conditions. GFAP^+^ astrocytes displayed ramified morphology without the long and thin processes that typically run in parallel to neuronal bundles in ND cultures (Fig. 3d and Supplementary Figure 3c). Of note, the neuronal and astroglial morphology in MLD-ARSA cultures suggested accelerated differentiation driven by ARSA overexpression, in line with IF and WB data. ARSA deficiency and ARSA overexpression did not apparently impact on the oligodendroglial morphology (Supplementary Figure 4).

These results suggest that ARSA deficiency affects oligodendroglial differentiation and maturation of human iPSC-NPCs. In addition, our time-course qualitative and quantitative analysis highlighted MLD-specific early defects in neuronal and astroglial differentiation that were previously overlooked or poorly elucidated in murine models.

### Impact of ARSA overexpression on glycosphingolipid biosynthesis and degradation

Altered expression of glycosphingolipid biosynthetic enzymes may impact on glial cell survival/differentiation^1^. Thus, we assessed the expression of CGT, GALC, and GAL3ST1, the enzymes that together with ARSA regulate the metabolism of sulfatide, galactosylceramide, and ceramide (Supplementary Figure 5a). Apart from reduced ARSA mRNA expression in MLD cultures, we found comparable expression of the other enzymes (Supplementary Figure 5b**)**. Interestingly, the supraphysiological ARSA mRNA expression in MLD-ARSA NPCs and differentiated progeny (Supplementary Figure 3a**)** was accompanied by increased expression of CGT, GALC, and GAL3ST1 enzymes, particularly at the late time points of differentiation (Supplementary Figure 5b).

These data suggest that ARSA overexpression impacts at the transcription level on the expression of some of the biosynthetic/catabolic enzymes regulating the metabolism of bioactive sphingolipids that are known regulators of cell homeostasis^40^.

### Sulfatide storage and altered sulfatide composition in MLD NPCs and differentiated progeny

**Fig. 4:**
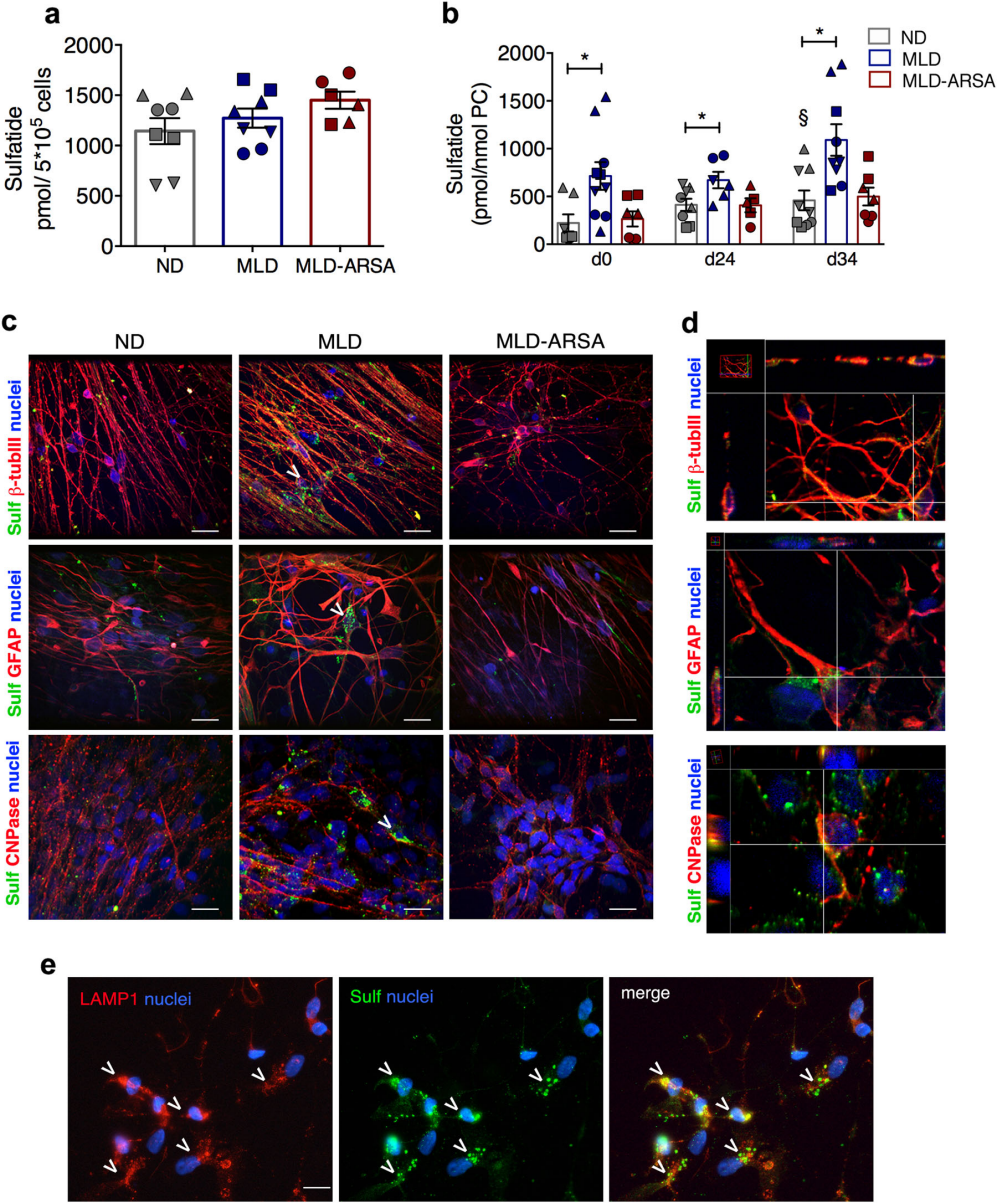
Progressive sulfatide storage in MLD iPSC-derived neural progeny. **a** Graph showing the total sulfatide content (pmol/5×10^5^ cells) in ND, MLD, and MLD-ARSA iPSCs. Data are expressed as the mean ± SEM; *n* = 3-4 iPSC clones/group in duplicate. Clones used: ND 1.1 (O); ND 1.3 (□); ND 2.2 (Δ); ND 2.3 (∇); MLD 1.1 (O); MLD 1.2 (□); MLD 1.3 (Δ); MLD 2.1 (∇); MLD-ARSA 1.1 (O), MLD-ARSA 1.2 (□); MLD-ARSA 1.3 (Δ). **b** Graph showing the total sulfatide content in ND, MLD, and MLD-ARSA cultures at different days of differentiation (d0, d24, d34). Sulfatide levels (pmol) are normalized to phosphatidylcholine (PC) levels (nmol). Data are expressed as the mean ± SEM; *n* = 4–6 independent experiments, 3–4 clones/group in duplicate. Clones used: ND 1.1 (O); ND 1.3 (□); ND 2.2 (Δ); ND 2.3 (∇); MLD 1.1 (O); MLD 1.2 (□); MLD 1.3 (Δ); MLD 2.1 (∇); MLD-ARSA 1.1 (O), MLD-ARSA 1.2 (□); MLD-ARSA 1.3 (Δ). Data were analyzed by one-way ANOVA followed by Tukey’s multiple comparisons test (for Gaussian-distributed values) and Kruskal−Wallis test (for non-Gaussian-distributed values). *p < 0.05; §*p* < 0.05 vs. ND d0. **c** Representative immunofluorescence confocal merged pictures showing sulfatide (Sulf, green) expression in neurons (β-tubulin III, red), astrocytes (GFAP, red), and oligodendrocytes (CNPase, red) in ND 2.3-, MLD 1.1- and MLD-ARSA 1.1-derived cultures (d24 of differentiation). Nuclei counterstained with DAPI (blue). Scale bars, 30 µm. Arrowheads identify neurons, astrocytes, and oligodendroglial cells expressing Sulf. **d** Representative confocal merged pictures after z-stack analysis showing co-expression of sulfatide (Sulf, green) and β-tubulin III (red, neurons), GFAP (red, astrocytes), and CNPase (red, oligodendrocytes) in clone MLD 1.1. Nuclei counterstained with DAPI (blue). Side views show the *xz* and *yz* planes of the stack. **e** Representative immunofluorescence pictures showing sulfatide expression (Sulf, green) in lysosomes (LAMP1, red) of MLD cells (d24 of differentiation; clone MLD 1.1). Arrowheads identify cells in the fields. Nuclei counterstained with DAPI (blue). Scale bar, 10 µm

We measured sulfatide content (20 sulfatide species) and composition (percentages of the different species on the total sulfatide) in ND, MLD, and MLD-ARSA iPSCs, NPCs (d0) and differentiated progeny (d24 and d34) by ultra-performance liquid chromatography-tandem mass spectrometry^41^. All iPSC populations displayed similar total sulfatide content (Fig. 4a and Supplementary Table 2). Sulfatide content increased up to threefold between d0 and d34 in ND cultures, in line with NPC differentiation and maturation (Fig. 4b). MLD iPSC-NPCs stored 2−3 times more sulfatide as compared to ND cells at all time points (Fig. 4b) and sulfatide levels were normalized in MLD-ARSA cultures (Fig. 4b and Supplementary Table 2). Of note, IF analysis revealed sulfatide storage in the soma and processes of MLD oligodendrocytes, neurons, and astrocytes that are not present in ND cells and are strongly reduced in MLD-ARSA cells (Fig. 4c, d). Co-localization of sulfatide and LAMP1-positive signal suggested lysosomal sulfatide storage (Fig. 4e).

**Fig. 5:**
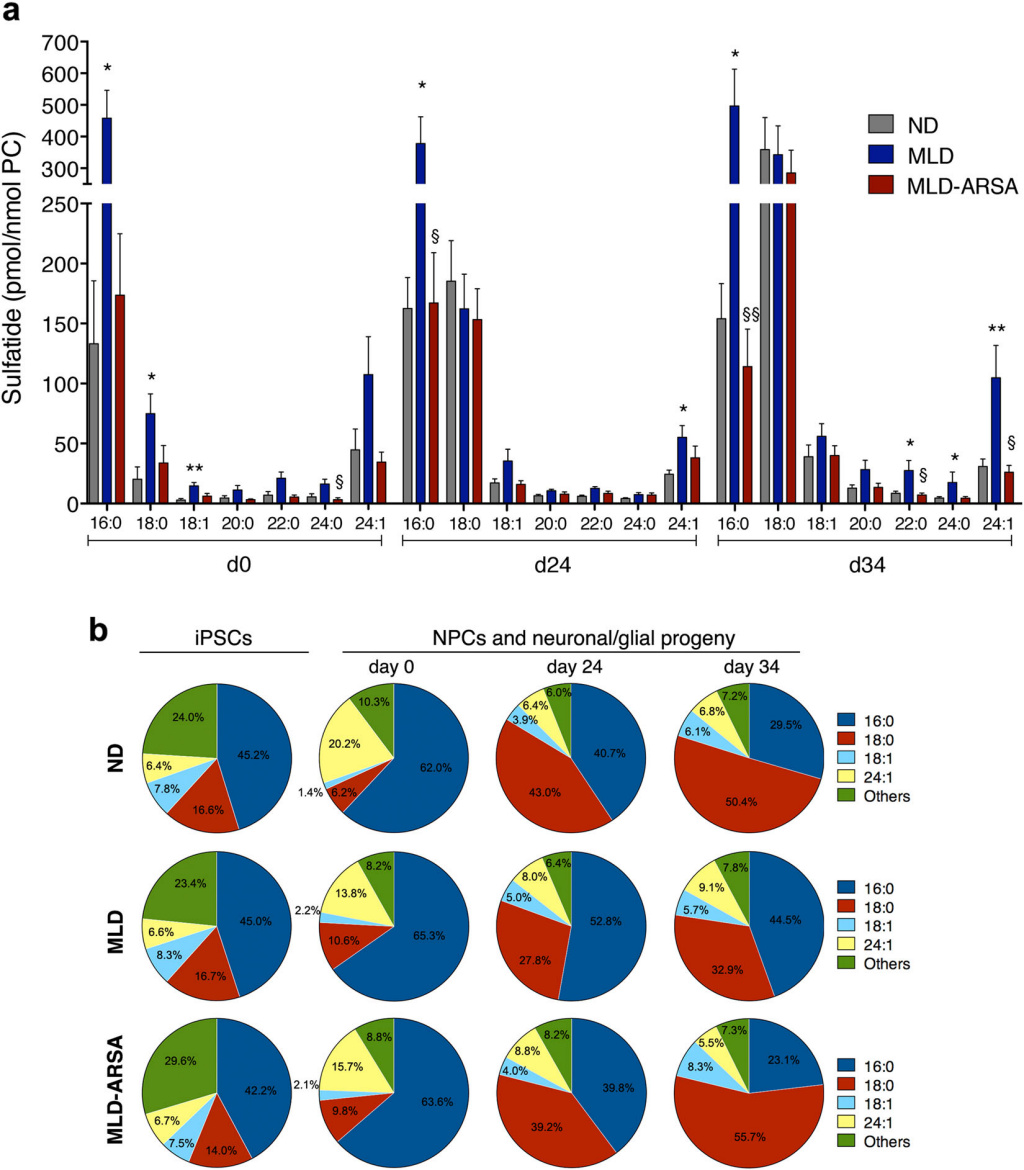
Sulfatide storage in MLD iPSC-derived neural progeny. **a** Bar graph showing the content of selected sulfatide species in ND, MLD, and MLD-ARSA cultures at different days during differentiation (d0, d24, d34). Data are expressed as the mean ± SEM; *n* = 4−6 independent experiments in duplicate, 3 clones/group with 1–3 replicates/clone. Clones used: ND 1.1, ND 1.3, ND 2.3, MLD 1.1, MLD 1.3, MLD 2.1, MLD-ARSA 1.1, MLD-ARSA 1.2, MLD-ARSA 1.3. Data related to each sulfatide species were analyzed by one-way ANOVA followed by Tukey’s multiple comparisons tests (for Gaussian-distributed values) and Kruskal−Wallis test (for non-Gaussian-distributed values) *p < 0.05 and **p < 0.01 vs. ND; §*p* < 0.05 and §§*p* < 0.01 vs. MLD. **b** Pie charts showing sulfatide composition in ND, MLD, and MLD-ARSA iPSCs, iPSC-NPCs and neuronal/glial progeny at different days of differentiation (d0, d24, d34). Values represent the percentage of single sulfatide species over the total sulfatide in each experimental group. Percentages of four selected sulfatide species are highlighted (16:0, 18:0, 18:1, 24:1); percentages of the remaining 16 species analyzed are pooled under the category “Others”. Data are expressed as the mean ± SEM; *n* = 4–6 independent experiments in duplicate, 3 clones/group with 1–3 replicates/clone. Clones used ND 1.1, ND 1.3, ND 2.3, MLD 1.1, MLD 1.3, MLD 2.1, MLD-ARSA 1.1, MLD-ARSA 1.2 MLD-ARSA 1.3

The C16−C18 sulfatide species were enriched in iPSC-derived cultures (Fig. 5a; Supplementary Table 2) and displayed a relevant increase (2–3 fold) during differentiation; they were 2–3 times more abundant in MLD as compared to ND cultures at all time points and were normalized in MLD-ARSA cells (Fig. 5a; Supplementary Table 2).

All NPC populations displayed neural-like sulfatide composition^20^ that was different from that of the correspondent iPSCs (Fig. 5b). ND cultures showed a time-dependent increase in C18:0 (≈6% and ≈50% at d0 and d34, respectively) at the expenses of C16:0 (≈60% and ≈30% at d0 and d34, respectively). In contrast, MLD cells maintained lower percentages of C18:0 (≈30%) and higher percentages of C16:0 (≈ 50%) at d34 as compared to ND cells **(**Fig. 5b**)**.

In summary, the little sulfatide load in MLD iPSCs suggests that primary storage is not the direct cause of the lysosomal expansion and intracellular ROS production observed in this cell type. In contrast, the pluripotent to neural transition is accompanied by a significant increase of sulfatide content, which may account for and contribute to lysosomal dysfunction and oxidative stress observed in MLD NPCs. Finally, the progressive sulfatide storage and abnormal sulfatide composition may contribute to the defective glial and neuronal differentiation from NPCs observed in MLD cultures.

### iPSC-derived neural cultures recapitulate early steps of MLD neurodegeneration

We sought to evaluate the appropriateness of the human iPSC-culture system to model the sulfatide storage and the defective neuronal and glial differentiation in MLD by a direct comparison with autoptic brain tissues of ARSA-deficient MLD patients and age-matched healthy donors (Supplementary Figure 6a). The total sulfatide storage in white matter brain tissues of MLD patients (Supplementary Figure 6b) was comparable to that found in MLD iPSC-derived differentiated neural progeny (Fig. 5b), even if the sulfatide species were differently represented. Isoforms with *C* ≥ 24 or with hydroxyl groups were predominant in human brain tissues, despite a significant reduction observed in MLD as compared to ND samples (Supplementary Figure 6c−d**)**.

The reduced expression of CNPase and β-Tubulin III proteins found in patient’s tissues (Supplementary Figure 6e−f) likely reflects the extensive oligodendroglial loss and neurodegeneration that characterize the late stages of MLD pathology as compared to the early pathological events captured using the iPSC-based system. Similarly, the increased GFAP expression in patient’s autoptic tissues (as compared to decreased/reduced expression in MLD iPSC cultures) is a sign of the extensive neuroinflammation associated to tissue damage.

These data highlight the relevance of the proposed iPSC-based model in detecting MLD-specific early defects during neuronal and astroglial development that cannot be properly assessed in CNS tissues of patients at the late stages of the disease.

## Discussion

The attainment of appropriate cell types that recapitulate the progression of CNS pathology characterizing neurodegenerative LSDs is required to develop a human disease model. Human iPSC-derived cultures highly enriched in neurons are used to model the neuronal defects of mucopolysaccharidosis^42–44^, Niemann−Pick type C^45–47^, Gaucher disease^48–51^, GM1 gangliosidosis^52^, and NCL^53^. On the other hand, nearly pure iPSC-derived glial progenitors and oligodendrocytes are used to model genetic demyelinating diseases^54,55^ and to reveal a causal role of glia in neurological disorders^56,57^. Here, we differentiated iPSCs^32^ into heterogeneous mixed populations of oligodendrocytes, neurons, and astrocytes. This allowed us monitoring the development of the main CNS cell types in a single-culture system and in a comprehensive time course analysis.

The lysosomal expansion, Golgi abnormalities, accumulation of endo-lysosomes, and increased ROS production described in our cellular model are in line with previous studies describing human iPSC-based models of LSDs^43,53,58^. Importantly, we highlight cell type- (iPSC vs. neural cells) and stage-specific (NPCs vs. differentiated progeny) tolerability to enzymatic deficiency and toxic effects of sulfatide that likely account for the time-dependent progression of disease pathology. The mild elevation of LAMP1 expression and ROS levels in MLD iPSCs was not associated with detectable changes in sulfatide content and composition. Sulfatide metabolism has not been investigated in pluripotent cells but may play a role in protein trafficking, cell adhesion, and cell-to-cell communication^1,59^. The neural conversion of iPSCs triggered the acquisition of a neural-specific sulfatide composition^20^ and promoted sulfatide storage in ARSA-deficient NPCs. Lack of important cell death and functional impairment in MLD iPSC-NPCs despite the presence of lysosomal expansion might relate to the GF-dependent proliferative state of these cells and, possibly, to the proposed intrinsic tolerability of iPSC-NPCs to toxic storage^60,61^. The differentiation of MLD iPSC-NPCs into oligodendrocytes, neurons, and astrocytes exacerbated sulfatide storage and cell dysfunction, highlighting significant abnormalities at the morphological, molecular, and biochemical level in the three cell lineages. The increase of ARSA activity and total sulfatide measured in ND neuronal and glial progeny as compared to their NPC counterpart resembles the physiological ARSA increase during CNS development and oligodendroglial differentiation^17^. Interestingly, we showed similar sulfatide storage in MLD iPSC-derived neural cells and autoptic white matter CNS tissues from MLD patients^62^. The prevalence of short-chain fatty acid sulfatide (C16, C18) in iPSC-derived neural cultures is likely related to the presence of relevant percentages of non-myelinating oligodendrocytes, neurons, and astrocytes^11,13,26^, in which these species are abundant^10^. Interestingly, we show an enrichment of sulfatide with short-chain fatty acid (<C24) in the white matter of MLD patients as compared to unaffected individuals, in line with the loss of mature myelin-forming cells. Despite the low proportion of >C24 sulfatide species in the iPSC-derived cultures, their increase at the late stages of differentiation is in accordance with their known expression later in oligodendrocyte development. Moreover, the disease-specific storage recapitulates what observed in white matter tissues of MLD patients.

The role of excessive sulfatide in delaying oligodendroglial differentiation/maturation has been investigated in murine systems^17–21^. Here we contribute to clarify this pathogenic mechanism in MLD patient-specific oligodendroglial cells. We show that ARSA deficiency impacts on sulfatide composition beyond sulfatide load. We envisage that the unbalanced C18/C16 ratio might favor the maintenance of immature oligodendroglial progenitors and impair their differentiation. Indeed, CNPase expression is reduced and delayed in MLD cultures during differentiation, while PDGFRα fails to be properly downregulated. The involvement of PDGFRα downstream signaling cascades (e.g. AKT pathway)^20^ in determining this phenotype remains to be investigated.

The impact of sulfatide accumulation on astroglial differentiation/maturation has been poorly investigated. A direct involvement of neuronal sulfatide storage to MLD pathology derives from clinical observations in MLD infantile patients^16^ and from studies describing axonal degeneration and neuronal dysfunction in MLD murine models^22–24^. Our data showing reduced and delayed neuronal and astroglial differentiation from MLD NPCs strongly suggest that sulfatide storage in the early stages of human CNS development might contribute to MLD neuropathology. We are currently exploring the mechanisms underlying the early and persistent alteration of neurite organization observed in MLD neurons (e.g. axonal loss and/or decreased microtubule stability)^63,64^. In our cultures, the flawed neuronal development is accompanied by aberrant morphology and delayed/reduced differentiation of astrocytes, features that have never been described in murine systems. Astrocytes cooperate with neurons on several levels, including formation, maintenance and elimination of synapses; neurotransmitter trafficking and recycling; ion homeostasis; energy metabolism; defense against oxidative stress^65^. These dynamic cell-to-cell interactions are shaped early in CNS development. Therefore, their disruption might contribute to MLD pathology before overt oligodendroglial cell death and neurodegeneration, with important implications in terms of timing and efficacy of treatments. Additional studies are needed to highlight the mechanism(s) underlying the disease-specific defects in astrocytes and in astrocyte-neuron-oligodendrocytes communication and to dissect the potential cause/effect relationships.

We provide proof of concept that normalization of sulfatide levels and composition achieved by LV-mediated ARSA overexpression in MLD iPSCs and neural progeny largely stabilizes the pathological cascade secondary to primary storage, rescuing the cell morphology and developmental expression pattern of glial and neuronal proteins. We also show that stable ARSA overexpression in MLD NPCs and differentiated progeny results in upregulation of GALC, CGT, and GAL3ST1 expression, suggesting an attempt of ARSA-overexpressing MLD cells to cope with the increased production of intermediate products consequent to enhanced sulfatide catabolism (i.e. galactosylceramide and ceramide, known to promote cell differentiation and apoptosis)^66^. Further studies are needed to assess how and to which extent glycosphingolipid homeostasis impacts on human NPC differentiation in a physiological vs. pathological background, and if such a mechanism contributes to the enhanced neuronal differentiation that we observed in MLD-ARSA cultures. Besides being important from a biological standpoint, this knowledge has important therapeutic implications. The iPSC-based system described here could represent a unique model to fully assess the safety of supraphysiological ARSA expression in human CNS cells in the context of gene therapy settings, complementing the studies performed so far in mice and large animals^67–72^ and providing crucial information for designing safe and effective CNS-directed gene therapy approaches.

## Experimental procedures

### Study design

In vitro experiments were planned and executed in the context of controlled laboratory experiments and with the help of expert staff if required (e.g. confocal analysis and z-stacks; Lysotracker assay). For each of the indicated experiments/assays we performed: (i) multiple (minimum of two) experimental and biological replicates and (ii) multiple (minimum of two) independent experiments, in order to generate an adequate number of samples in each group. The experimental sample size was determined on the basis of previously collected data in the same experimental settings and by the interim analysis of results. No data were excluded from the analysis, unless in the presence of a clear technical flaw (e.g. incomplete transfer of protein on nitrocellulose membranes; unexpected results in the positive/negative controls included in the experiments). Investigators conducting the experiments were not blinded to sample code. Investigator processing samples at the end of the experiment were blinded to sample code in some experimental settings (e.g. confocal picture acquisition from stained coverslips and quantification of cell type composition) up to the stage of data analysis. For experimental details, please refer to the dedicated sections below and in the Supplementary Materials.

### iPSC lines

The normal donor (ND) and patient-specific (MLD) iPSC lines used in this study as well as the generation of ARSA-overexpressing MLD iPSCs by LV-mediated gene transfer have been previously described^32^. The source and characteristics of the original ND and MLD fibroblast cell lines, as well as the list of ND, MLD, and MLD-ARSA iPSCs clones used in this study, is reported in the Supplemental Experimental Procedures.

### Cell culture

iPSC culturing and expansion has been performed as described in the Supplemental Experimental Procedures. For neural induction, ND, MLD, and MLD-ARSA hiPSC colonies were detached with Accutase (Life Technologies), suspended as single cells and plated on Matrigel (BD Pharmingen)-coated dishes (50,000 cells/cm^2^) in MEF pre-conditioned iPSC medium (IPSCM) in the presence of 10 µM of the Rho kinase inhibitor Y-27632 (Sigma). When the cell culture reached ≈90% confluence (usually 2 days after plating), culture medium was replaced with KSR medium (KSRM) supplemented with 200 ng/ml of rhNOGGIN (R&D) and 10 µM of SB431542 (Sigma). The medium was changed daily for the next 3 days. Thereafter, it was switched every other day in order to gradually expose the cells to increasing (1:3, 1:1, 3:1) ratio of NPC/KSR medium. 2 days after the last switch the cells were detached using Accutase and plated on Matrigel-coated dishes in NPCM supplemented with 20 ng/ml bFGF and 20 ng/ml EGF. In these conditions, cells proliferated for a limited number of passages. For all the differentiation experiments we used cells between p2 and p4. Cells were detached using Accutase and plated on Matrigel-coated dishes (20,000 cells/cm^2^) in NPCM (d0). During the first 4 days after plating (d4) we gradually replaced NPCM containing bFGF and EGF with increasing amount of GDM, supplemented with 10 ng/ml PDGF-AA, 10 ng/ml NT3, 10 ng/ml IGF-1, 5 ng/ml HGF, and 60 ng/ml T3. From d4 to d13 medium was changed every other day. From d14 to d23, PDGF-AA, NT3, IGF-1, and HGF were withdrawn from the medium (Glial maturation medium GMM). At d24, the medium was switched to GMM supplemented with 20 µg/ml of ascorbic acid (Sigma) until d34. For details on culture media, see the Supplemental Experimental Procedures.

### Human tissues

Post-mortem snap-frozen brain samples from four MLD patients and four age-matched healthy donors were obtained from the NIH NeuroBioBank. For details see the Supplemental Experimental Procedures.

### ROS analysis

Reactive oxygen species were detected using CellROX^®^ Deep Red Reagent (Thermo Scientific). For details, see the Supplemental Experimental Procedures.

### Image acquisition and analysis

Samples were visualized with Nikon Eclipse Ni microscope using double laser microscopy with Nikon Plan- Fluor (×20 -air and ×40 -air magnification) objective lens (Nikon, Monacalieri, Italy). Images were acquired using a camera (Nikon DS Fi2) and NIS-Element F4.00.00 acquisition software (Nikon). Immunopositive area of acquired images (expressed in pixels) was calculated using the ImageJ software. ND samples were used to set the threshold. The immunopositive area was normalized to the number of nuclei in the same field. At least 10 fields/sample with 20–30 cells/field were quantified in *n* = 3 experiments in triplicate.

### Confocal and Z-stack

Co-labeling between sulfatide (Sulf) and differentiation markers (β-tubulin III, GFAP,  CNPase) was analyzed in differentiated cultures. Z-stacks were recorded utilizing a PerkinElmer UltraVIEW ERS Spinning Disk Confocal (PerkinElmer Life Sciences, Inc.) using a ×63/NA1.4 Plan-Apochromat oil-immersion lens (Carl Zeiss, Jena, Germany) with a 405 nm diode laser, 488 nm argon laser and a 568 nm krypton laser excitation wavelengths. Sequential confocal images were collected at 0.25 μm intervals covering 14 μm depth (56 total scanning images for each channel). The 3D signals and the orthogonal projection representations were obtained by means of Volocity Software (v5.2.1; PerkinElmer-Improvision, Lexington, MA, USA). Images were imported into Adobe Photoshop CS4 (USA) and adjusted for brightness and contrast.

### Analysis of sulfatide content

Analysis of sulfatide content was performed as previously described^41^. For details see Supplemental Experimental Procedures.

### Statistical analysis

In vitro cell counts, data from qRT-PCR, western blot, mass spectrometry, enzyme activity, and data obtained following the quantification of the immunopositive area by the ImageJ software were analyzed with Graph Pad Prism 6.0c for Macintosh and expressed as the mean ± standard error of the mean (SEM, with *n* ≥ 3). Unpaired *t* test (statistical significance: *p* < 0.05) was used to compare two independent groups. One-way ANOVA (followed by Bonferroni post-test) or Kruskal−Wallis test (followed by Tukey’s or Dunn’s post-test) were used for statistical analyses of more than two independent groups in the presence of Gaussian or non-Gaussian distribution of data (assessed by Kolmogorov−Smirnov test), respectively. The number of samples and the statistical test used are indicated in the figure legends.

## Electronic supplementary material

Supplemental material
