# Supplementary material for: Human iPSC-based models highlight defective glial and neuronal differentiation from neural progenitor cells in metachromatic leukodystrophy
Source: Cell Death Dis. 2018 Jun 13;9(6):698. doi: 10.1038/s41419-018-0737-0 (PMC5997994; doi:10.1038/s41419-018-0737-0)
Supplement: Supplementary file 1 — Supplemental material [file 41419_2018_737_MOESM1_ESM.pdf]

Supplemental information

- **Supplementary tables and figures**
  - Supplementary Table 1
  - Supplementary Table 2
  - Supplementary Figure 1
  - Supplementary Figure 2
  - Supplementary Figure 3
  - Supplementary Figure 4
  - Supplementary Figure 5
  - Supplementary Figure 6

- **Supplementary methods**
  - Induced pluripotent stem cells lines
  - Cell culture
  - Lentiviral-mediated gene transfer
  - Human brain tissues
  - Gene expression studies
  - Immunofluorescence
  - ROS analysis
  - Lysotracker analysis
  - Electromicroscopy analysis
  - Western Blot
  - Analysis of sulfatide content
  - Quantification of ARSA Activity
  - Microelectrode Array (MEA) experiments and analysis

- **Supplementary References**

Supplementary Tables and Figures

Supplementary Table 1 (related to Figure 3). Cell type composition of ND, MLD and MLD-ARSA iPSC-NPCs and differentiated progeny at different days in culture.

Percentage of cells immunopositive for markers of neuroepithelial cells (NESTIN), neuronal cells (b-tubulin III), astroglial cells (GFAP) and oligodendroglial cells (A2B5, NG2, O4, CNPase, APC, OLIG2) on total number of cells assessed by immunofluorescence assay and confocal analysis in ND, MLD and MLD-ARSA cultures at different time points (days, d) during differentiation. Data are expressed as the mean ± SEM; n=6 (ND and MLD) and n=4 (MLD-ARSA) independent experiments; 3 clones/group analyzed. Clones used: ND 1.1; ND 1.3; ND 2.2; MLD 1.1 MLD 1.3; MLD 2.1; MLD-ARSA 1.1; MLD-ARSA 1.2; MLD-ARSA 1.3.

	**NPCs and differentiated neuronal/glial progeny Days in culture (d)**								
	**d0**			**d14**			**d24**		
	**ND**	**MLD**	**MLD-ARSA**	**ND**	**MLD**	**MLD-ARSA**	**ND**	**MLD**	**MLD-ARSA**
**NESTIN**	76.5 ± 4.5	61.8 ± 9.2	76.5 ± 3.5	10.0 ± 3.3	7.4 ± 2.1	6.1 ± 2.0	2.6 ± 0.5	6.0 ± 1.6	5.7 ± 0.7
**A2B5**	15.6 ± 7.6	25.7 ± 9.9	11.2 ± 3.6	22.3 ± 6.1	22.3 ± 0.7	30.0 ± 3.9	6.2 ± 0.3	10.3 ± 1.3	4.4 ± 0.1
**NG2**	7.8 ± 1.6	12.5 ± 5.3	12.5 ± 3.3	2.2 ± 1.9	0.7 ± 0.4	2.8 ± 0.2	1.2 ± 0.0	0.8 ± 0.4	1.5 ± 0.2
**OLIG2**	0	0	0	4.9 ± 1.7	11.4 ± 2.8	8.5 ± 1.1	8.7 ± 2.2	10.8 ± 1.9	6.9 ± 0.1
**O4**	0	0	0	1.8 ± 1.0	4.0 ± 2.7	1.7 ± 1.7	8.5 ± 2.6	7.4 ± 2.2	6.7 ± 0.3
**CNPase**	0	0	0	20.3 ± 4.8	25.2 ± 7.0	18.5 ± 6.4	27.8 ± 1.5	23.9 ± 1.7	27.0 ± 1.8
**APC**	0	0	0	9.2 ± 2.3	9.0 ± 1.3	4.8 ± 1.1	13.7 ± 3.7	15.6 ± 5.3	15.0 ± 0.7
**GFAP**	0	0	0	4.2 ± 1.0	3.7 ± 1.0	2.4 ± 0.1	4.9 ± 1.6	2.7 ± 1.1	4.9 ± 1.6
**β-tubulin III**	2.5 ± 0.1	3.6 ± 0.1	3.9 ± 0.2	11.8 ± 2.1	8.1 ± 1.1	14.6 ± 2.8	11.5 ± 2.6	9.0 ± 0.4	26.5 ± 4.3

Supplementary Table 2 (related to Figure 4 and Figure 5). Sulfatide content of ND, MLD and MLD-ARSA iPSCs, iPSC-NPCs and differentiated progeny.

The table reports the quantification of all sulfatide species analyzed by UPLC-MS/MS (Blomqvist et al., 2017) in ND, MLD and MLD-ARSA iPSCs, iPSC-NPCs and neuronal/glial progeny at different time points (days, d) during differentiation (d0, d24, and d34). Data are expressed as pmol of sulfatide/5*10^5 cells (iPSCs) and pmol of sulfatide/nmol of phosphatidylcholine (NPCs and progeny) and represent the mean±SEM; n=6 (ND and MLD) and n=4 (MLD-ARSA) independent experiments in duplicate; 3-4 clones/group**.** Clones used: ND 1.1, ND 1.3, ND 2.2, ND 2.3, MLD 1.1, MLD 1.2, MLD 1.3, MLD 2.1, MLD-ARSA 1.1, MLD-ARSA 1.2, MLD-ARSA 1.3.

	**iPSCs**			**NPCs and differentiated neuronal/glial progeny Days in culture (d)**								
				**d0**			**d24**			**d34**		
**Sulfatide species**	**ND**	**MLD**	**MLD-ARSA**	**ND**	**MLD**	**MLD-ARSA**	**ND**	**MLD**	**MLD-ARSA**	**ND**	**MLD**	**MLD-ARSA**
**16:0**	487.14 ± 63.31	525.61 ± 38.62	585.84 ± 43.71	133.15 ± 52.42	458.36 ± 88.12	173.46 ± 51.27	162.46 ± 25.65	378.06 ± 84.50	167.20 ± 41.88	154.02 ± 29.23	496.72 ± 116.64	114.12 ± 31.29
**18:0**	202.77 ± 47.90	231.24 ± 46.28	210.75 ± 5.00	20.28 ± 10.30	74.88 ± 16.42	33.90 ± 14.58	185.29 ± 33.81	162.26 ± 28.88	153.30 ± 25.65	358.65 ± 101.36	342.53 ± 91.39	284.97 ± 72.21
**18:1**	95.71 ± 22.72	115.60 ± 19.02	103.88 ± 1.71	3.06 ± 1.21	14.82 ± 2.71	6.22 ± 2.24	17.26 ± 3.43	35.43 ± 9.76	16.05 ± 2.94	39.15 ± 9.66	56.03 ± 10.50	40.02 ± 8.25
**20:0**	61.56 ± 11.50	67.75 ± 12.45	66.02 ± 3.41	4.68 ± 1.91	11.33 ± 3.76	3.27 ± 0.64	6.61 ± 0.99	10.81 ± 1.20	7.85 ± 1.82	12.83 ± 2.65	28.42 ± 7.74	13.43 ± 3.58
**22:0**	92.08 ± 17.11	93.53 ± 7.28	159.98 ± 38.22	7.08 ± 2.91	21.15 ± 5.20	5.61 ± 1.80	6.12 ± 0.90	12.66 ± 1.52	8.45 ± 1.81	8.60 ± 1.70	27.56 ± 8.39	7.18 ± 1.66
**24:0**	31.73 ± 5.56	33.10 ± 1.99	70.53 ± 30.33	5.57 ± 2.52	16.32 ± 3.95	3.45 ± 1.50	4.25 ± 0.63	7.50 ± 1.57	7.25 ± 1.67	4.66 ± 1.10	17.59 ± 8.73	4.60 ± 1.45
**24:1**	75.63 ± 13.81	86.92 ± 9.78	110.44 ± 20.70	44.85 ± 17.28	107.46 ± 31.56	34.45 ± 8.48	24.45 ± 3.41	55.12 ± 9.81	38.28 ± 9.78	30.83 ± 6.31	104.80 ± 26.99	26.07 ± 5.80
**25:1**	7.24 ± 1.30	7.27 ± 1.03	11.56 ± 2.44	0.18 ± 0.18	0	0.16 ± 0.10	0	0	0	0.11 ± 0.06	0.29 ± 0.20	0.15 ± 0.15
**26:0**	1.10 ± 0.21	1.06 ± 0.11	1.15 ± 0.14	0.05 ± 0.05	0.12 ± 0.05	0.03 ± 0.03	0.01 ± 0.01	0.01 ± 0.01	0.03 ± 0.03	0.01 ± 0.01	0.20 ± 0.12	0.02 ± 0.02
**26:1**	3.31 ± 0.54	3.41 ± 0.45	4.21 ± 0.67	1.13 ± 0.61	3.25 ± 0.83	0.59 ± 0.23	0.83 ± 0.20	1.39 ± 0.24	1.17 ± 0.30	0.89 ± 0.22	2.13 ± 0.60	0.81 ± 0.18
**16:0-OH**	0	0	0	0	0	0	0	0	0	0	0	0
**18:0-OH**	18.34 ± 5.46	25.12 ± 7.25	28.37 ± 1.93	0.40 ± 0.20	1.40 ± 0.52	0.44 ± 0.28	2.14 ± 0.33	3.99 ± 0.16	2.99 ± 0.25	4.34 ± 0.96	7.92 ± 1.17	4.25 ± 0.45
**20:0-OH**	4.18 ± 1.09	6.2 ± 3.01	5.56 ± 1.47	0.28 ± 0.19	0.34 ± 0.28	0.12 ± 0.07	0.07 ± 0.03	0.27 ± 0.04	0.18 ± 0.06	0.21 ± 0.11	0.07 ± 0.03	0.31 ± 0.08
**22:0-OH**	33.01 ± 7.26	39.74 ± 15.91	47.07 ± 9.84	0.60 ± 0.28	1.38 ± 0.35	0.68 ± 0.23	0.25 ± 0.05	0.45 ± 0.06	0.40 ± 0.10	2.43 ± 2.00	1.84 ± 0.61	0.23 ± 0.05
**23:0-OH**	10.83 ± 1.75	12.46 ± 3.86	14.32 ± 1.75	0.17 ± 0.12	0.66 ± 0.27	0.86 ± 0.42	1.34 ± 0.14	1.19 ± 0.09	1.89 ± 0.18	1.10 ± 0.25	0.88 ± 0.29	1.39 ± 0.30
**24:0-OH**	10.68 ± 2.16	13.62 ± 4.16	21.03 ± 2.77	0.11 ± 0.11	0.69 ± 0.38	0.14 ± 0.09	0.10 ± 0.05	0.47 ± 0.11	0.14 ± 0.06	0.64 ± 0.44	0.95 ± 0.83	0.26 ± 0.09
**24:1-OH**	6.70 ± 1.23	7.97 ± 2.51	8.30 ± 1.88	0.99 ± 0.35	1.41 ± 0.39	0.51 ± 0.25	0	0.19 ± 0.12	0.08 ± 0.08	1.86 ± 1.53	1.69 ± 0.67	0.51 ± 0.45
**25:0-OH**	1.35 ± 0.40	1.33 ± 0.92	1.72 ± 0.35	0	0	0	0	0	0.10 ± 0.10	0.04 ± 0.04	0	0.09 ± 0.06
**25:1-OH**	0	0	0	0.24 ± 0.15	0.71 ± 0.30	0.71 ± 0.34	1.46 ± 0.15	1.09 ± 0.08	1.71 ± 0.34	0.91 ± 0.20	0.57 ± 0.19	1.03 ± 0.24
**26:0-OH**	0	0	0	0.42 ± 0.18	0.49 ± 0.17	0.25 ± 0.12	0.01 ± 0.01	0.27 ± 0.25	0.02 ± 0.02	0.22 ± 0.11	0.50 ± 0.19	0.21 ± 0.17

Supplementary figure 1

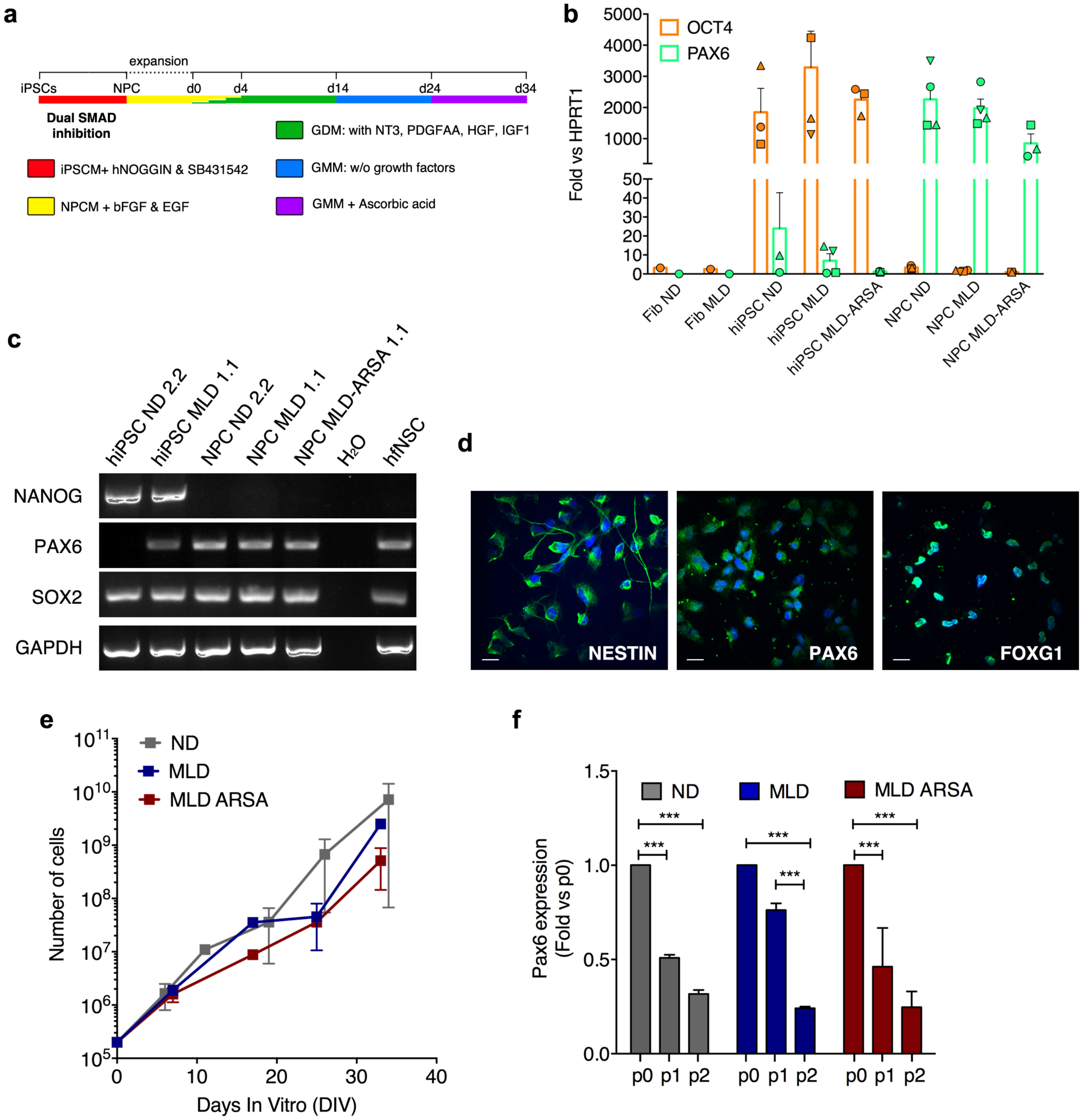

Supplementary Figure 1. Molecular and functional characterization of iPSC-NPCs.

(a) Schematic of the differentiation protocol used to obtain neuroepithelial progenitor cells (NPCs) and differentiated neuronal, astroglial and oligodendroglial progeny from ND, MLD, and MLD-ARSA iPSCs. Abbreviations: GDM: glial differentiation medium, bFGF: basic fibroblast growth factor; EGF: epidermal growth factor; GDM: glial differentiation medium; NT3: Neurotrophin 3; PDGF-AA: platelet-derived growth factor-AA; HGF: Hepatocyte growth factor; IGF1: Insulin-like growth factor 1; GMM: Glial maturation medium; d, days.

(b) Bar graph showing OCT4 and PAX6 mRNA expression levels (normalized on the HPRT1 gene) assessed by qRT-PCR in ND, MLD and MLD-ARSA iPSCs and iPSC-derived NPCs (ND 1.1 (O); ND 1.3 (□); ND 2.2 (△); ND

2.3 (▽); MLD 1.1 (O); MLD 1.2 (□); MLD 1.3 (△); MLD 2.1 (▽); MLD-ARSA 1.1 (O), MLD-ARSA 1.2 (□); MLD-ARSA 1.3 (△). Parental fibroblasts (Fib) were used as negative control. Data are expressed as mean ± SEM; n= 3-4 experiments in duplicate.

(c) Representative RT-PCR showing NANOG, PAX6, and SOX2 mRNA expression during the differentiation of ND, MLD and MLD-ARSA iPSCs in NPCs (clones specified in the panel). GAPDH was used as loading control. hfNSC, human fetal-derived neural stem cells (positive control) (Meneghini et al., 2017).

(d) Representative immunofluorescence merged pictures showing the expression of the neuroepithelial markers NESTIN, PAX6 and FOXG1 (green) in NPCs (clone ND 1.1). Nuclei are counterstained with DAPI (blue); scale bar: 20μm.

(e) Stable proliferation and expansion of ND, MLD and MLD-ARSA NPCs for 4-5 sub-culturing passages (30-35 days in vitro, DIV). Data are expressed as the mean ± SEM; n=2-3 NPC lines/group; clones used: ND 1.1, ND 1.3 ND 2.2; MLD 1.1, MLD 1.2, MLD 1.3; MLD-ARSA 1.1, MLD-ARSA 1.2).

(f) Progressive downregulation of PAX6 mRNA expression (normalized on p0) assessed by qRT-PCR in ND MLD and MLD-ARSA NPCs. Clones used: ND 1.1, ND 1.3 ND 2.2; MLD 1.1, MLD 1.2, MLD 1.3, MLD-ARSA 1.1, MLD-ARSA 1.2. p, passages in culture. Data are expressed as the mean ± SEM; n=2-3 NPC lines/group (as in e) ***p<0.001 Two-way Anova followed by Bonferroni's multiple comparison test.

Supplementary figure 2

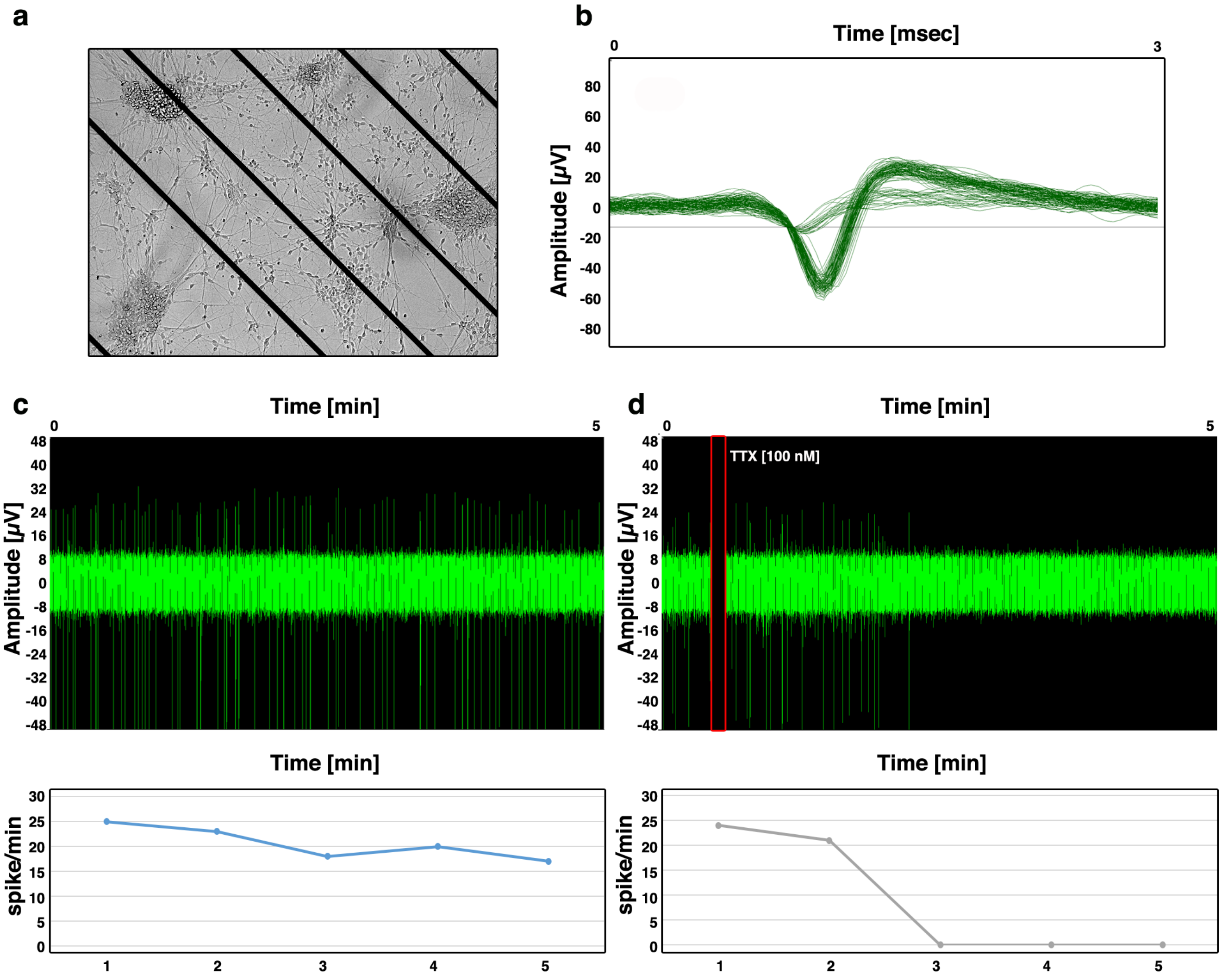

Supplementary Figure 2. Electrical properties of iPSC-derived neurons at d24 of differentiation.
(a) Phase contrast image of iPSC-derived differentiated neuronal-like cells plated on MEA chips. Black traces are electrical connections on the chip.
(b) Spikes recorded with MEA system in 5 minutes have been overlapped in order to show the waveforms (green traces). Note the presence of two types of spikes.
(c-d) Representative spiking activity in a single MEA channel under control condition (c) and after treatment with the voltage-dependent sodium channel blocker Tetrodotoxin (TTX) (d). Red box in (d) represents the time of drug injection. Graphs represent the average spike number per minute.
Data in a-d derive from clone ND 2.3

Supplementary figure 3

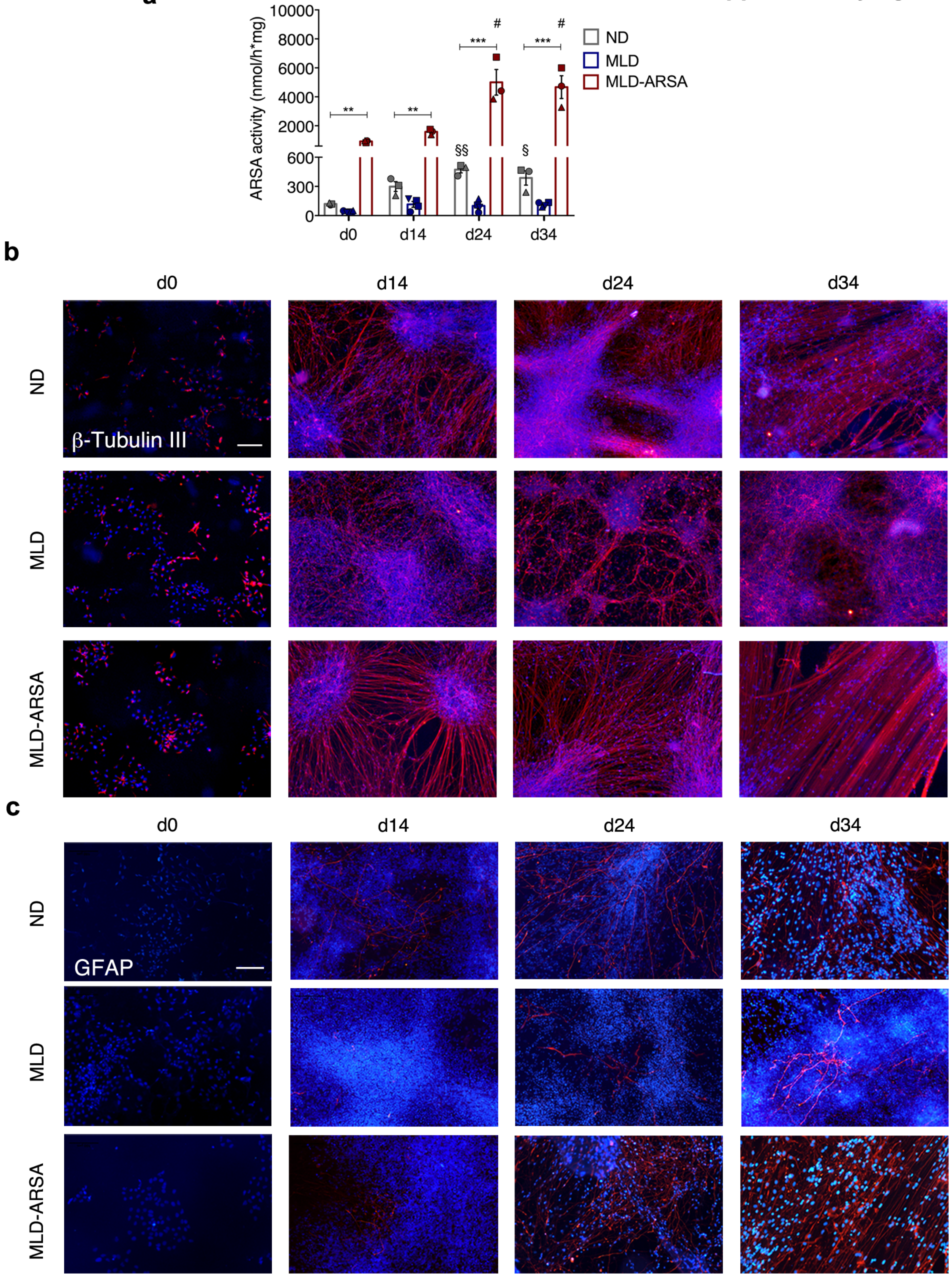

Supplementary Figure 3. Morphology, cellular organization and enzymatic activity of NPC-derived neuronal and glial progeny.

(a) Supraphysiological ARSA activity (measured using *p*-nitrocatechol assay) in ND, MLD, and MLD-ARSA NPCs (d0) and neuronal/glial progeny at different time points of differentiation (d14, d24, and d34). Data are expressed as the mean ± SEM; n=4 independent experiments. Clones used: ND 1.1, ND 1.3 ND 2.2; MLD 1.1, MLD 1.2, MLD 1.3, MLD 2.1, MLD-ARSA 1.1, MLD-ARSA 1.2, MLD-ARSA 1.3. Data from each time point (different groups) and data in each group (different time points) were analyzed by One-Way ANOVA followed by

Dunn’s multiple comparisons tests. **p<0.01; ***p<0.001; §p<0.05 and §§p<0.01vs ND d0; # p<0.001 vs MLD-ARSA d0.

(b, c) Representative immunofluorescence pictures showing morphology and cellular organization of neurons (b; β-tubulin III, red) and astrocytes (c; GFAP, red) in ND, MLD and MLD-ARSA cultures (clones ND 2.3, MLD 2.1 and MLD-ARSA 1.1) at different days of differentiation (d0, d14, d24, d34). Nuclei are counterstained with DAPI (blue). Scale bars: 50μm

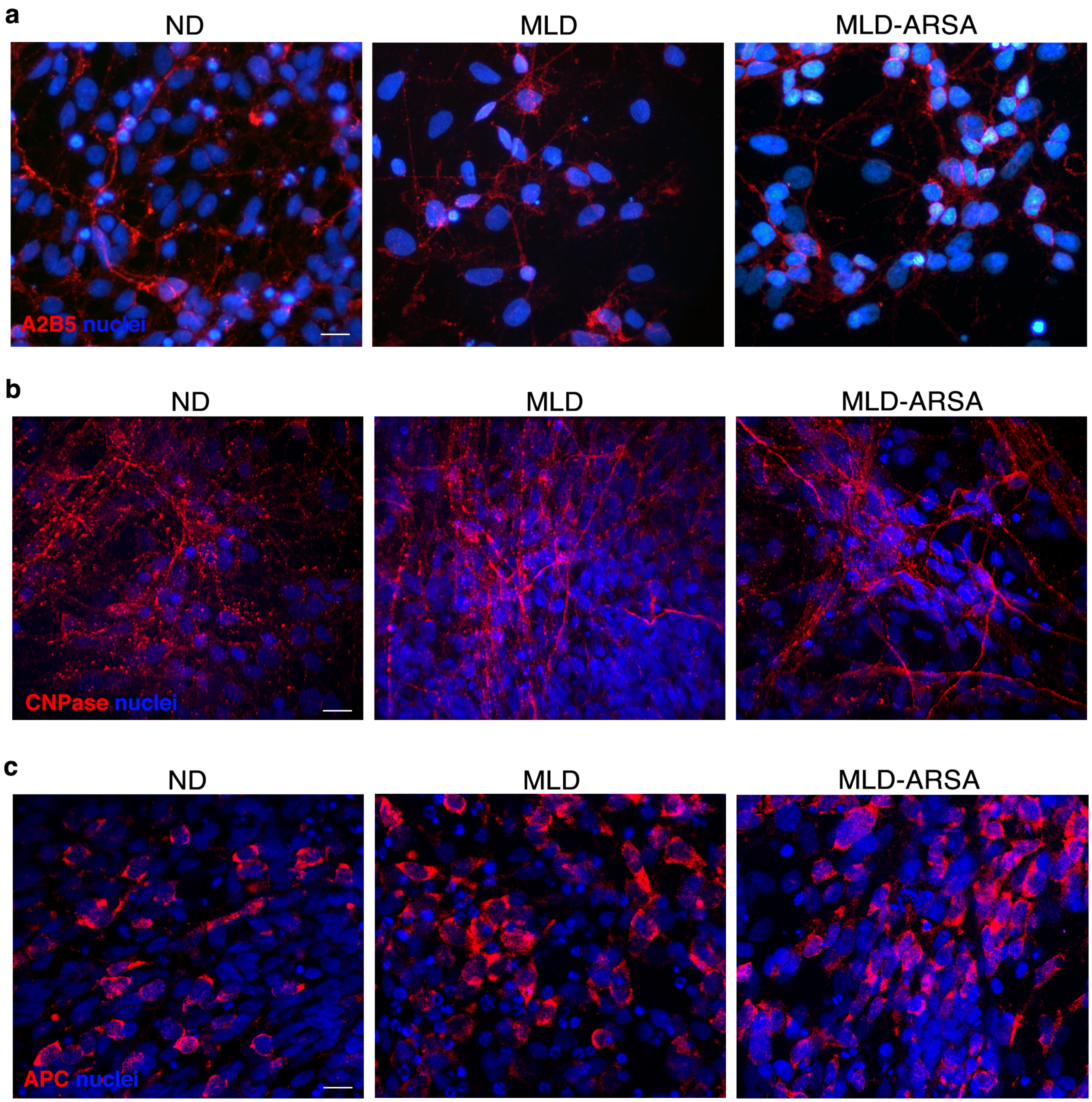

Supplementary Figure 4. Morphology and cellular organization of NPC-derived oligodendrocytes.
(a-c) Representative immunofluorescence pictures showing the morphology of oligodendrocyte progenitors (a; A2B5, red) and mature oligodendrocytes (b; CNPase, red c; APC, red) in ND, MLD and MLD-ARSA cultures (clones ND 2.3, MLD 2.1 and MLD-ARSA 1.1) at d24 of differentiation. Nuclei are counterstained with DAPI (blue). Scale bars: 20μm

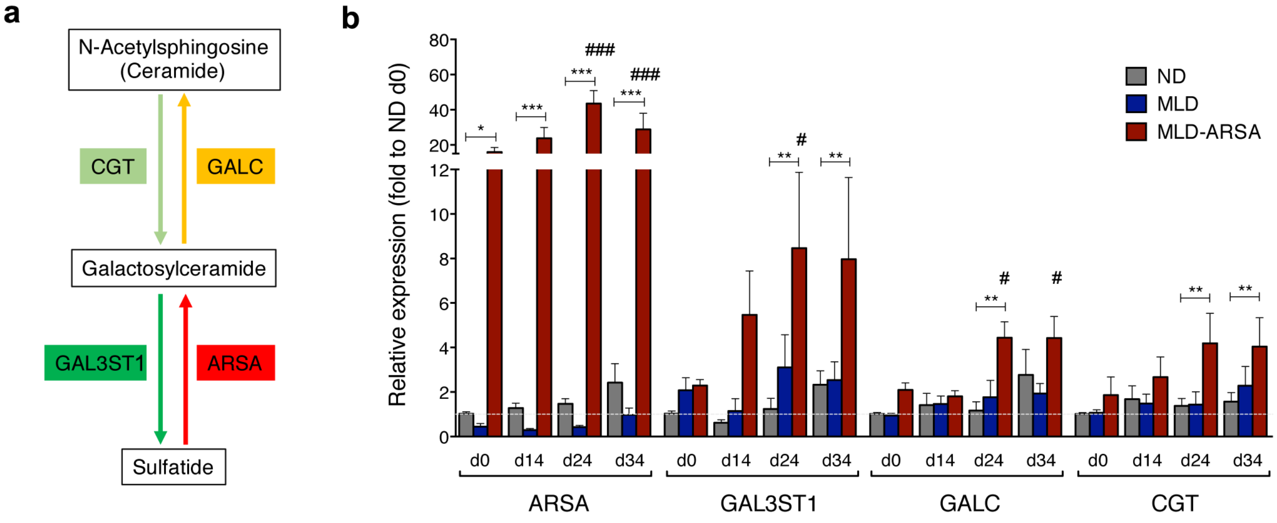

Supplementary Figure 5. Expression of enzymes involved in the sulfatide pathway in iPSC-derived neural progeny.

(a) Schematic representation of the enzymes involved in sulfatide synthesis and degradation. Abbreviations: CGT: UDP-Galactose-Ceramide Galactosyltransferase, GALC: Galactosylceramidase, GAL3ST: Galactose-3-O-Sulfotransferase (also known as cerebroside sulfotransferase, CST); ARSA: Arylsulfatase A.

(b) Relative mRNA expression of biosynthetic and catalytic enzymes in ND, MLD and MLD-ARSA iPSC-NPCs (d0) and differentiated progeny (d14, d24, and d34). Data are expressed as the mean ± SEM; n=3 experiments run in duplicate. 2 clones/group. Clones used: ND 1.3, ND 2.3, MLD 1.1, MLD 2.1, MLD-ARSA 1.1, MLD-ARSA 1.3. Data were analyzed by two-way ANOVA followed by Dunnett's multiple comparison test. *p<0.05, **p<0.01, ***p<0.001; #p<0.05, ###p<0.001 vs. MLD-ARSA d0.

.

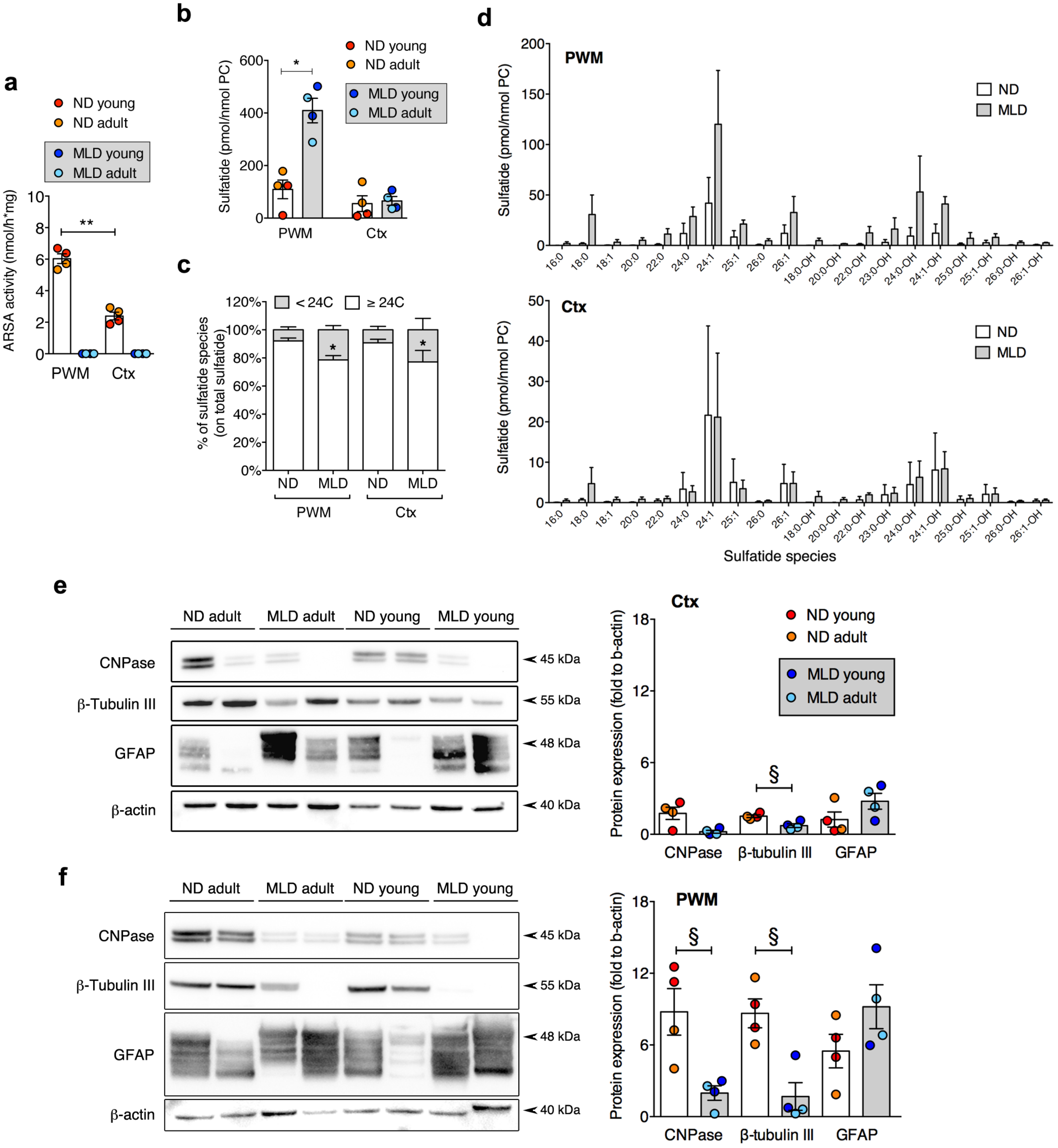

Supplementary Figure 6. Neuronal and glial cell loss and sulfatide storage in brain tissues of ND and MLD patients.

(a) Undetectable ARSA activity in autoptic brain tissues (periventricular white matter, PWM; cerebral cortex, Ctx) from 4 MLD patients of different age (young: 2-4 years; adult: 17-24 years) compared with physiological ARSA activity in region- and age-matched samples from 4 normal donors (ND). See also Supplementary Methods. Data are expressed as the mean ± SEM; n=1 experiment in duplicate (each dot represents the average of the two replicates). Data were analyzed by unpaired Student t-test; **p<0.01.

(b) Total sulfatide content in autoptic brain tissues (periventricular white matter, PWM; cerebral cortex, Ctx) of 4 MLD patients of different age (young: 2-4 years; adult: 17-24 years) determined by ultra-performance liquid chromatography-tandem mass spectrometry (UPLC-MS/MS)(Blomqvist et al., 2017). Sulfatide levels (pmol) are normalized to phosphatidylcholine (PC) levels (nmol). Data are expressed as the mean ± SEM; n=1 experiment in duplicate (each dot represents the average of the two replicates).

(c) Stacked column graph showing the relative percentage of sulfatide with short chain (C<24) and long-chain (C≥24) fatty acid in the periventricular white matter (PWM) and cerebral cortex tissues (Ctx) of ND (n=4) and MLD patients (n=4). Data analyzed by Unpaired Student t-test; *p<0.05 vs ND

(d) Bar graphs showing the levels of sulfatide species (C16 to C26) detected in the periventricular white matter (PWM) and cerebral cortex tissues (Ctx) of ND (n=4) and MLD patients (n=4). Data are expressed as the mean ± SEM; n=1 experiment in duplicate

(e-f) Representative Western Blots showing the expression of CNPase, β-tubulin III and GFAP proteins in the Ctx (b) and PWM tissues (c) derived from ND and MLD brains. Graphs show the quantification of WB bands after normalization of b-actin. Data are expressed as the mean ± SEM; n=1 experiment in duplicate (each dot represents the average of the two replicates). Data were analyzed by Unpaired Student t-test; §p<0.05.

Supplementary Methods

Induced pluripotent stem cells lines

The normal donor (ND) and patient-specific (MLD) iPSC lines used in this study have been previously described (Meneghini et al, 2017). In **Table A** we summarize the origin of the cell lines and the disease-causing mutation of MLD cell lines (including the disease variant and the allele's reference). ARSA mutations are reported according to current nomenclature guidelines, ascribing the A of the first ATG translational initiation codon as nucleotide +1 (http://www.hgvs.org/mutnomen) (Cesani et al., 2016). The traditional nomenclature that describes mutations based on the mature proteins (which differs from the translated protein in six nucleotides at the 5'-terminus of the cDNA sequence and two amino acids at the N-terminus) is reported for comparison. LI, late infantile. In **Table B** we report the ARSA-overexpressing MLD iPSC clones, including the untransduced (UT) MLD clone of origin, the therapeutic vector, and the ARSA activity (together with ARSA activity of ND and MLD clones)(Meneghini et al., 2017). Human cells were used according to the guidelines on human research issued by the San Raffaele Scientific Institute's ethics committee, in the context of the protocol TIGET-HPCT. All the cells used were mycoplasma-free (tested once every two weeks).

Table A

**ID**	**Fibroblasts** Code nr./ Source	**Age**	**Mutation** Traditional nomenclature	**Mutation** Current nomenclature	**Protein/RNA**	**Disease variant**	**Allele's Refs**
MLD1	FFF0951989/ Gaslini Biobank	6 years	[459+1G>A] + [1216del9 1049A>G/1620A>G]	[c.465+1G>A] + [c1223_1231del9 c.1055A>G/c*96A>G]	Splicing site in intron 2 + deletion (p.Ser406_Thr408del) N352S/pseudodeficiency	LI	(Berger et al., 1999) (Polten et al., 1991) (Nelson et al., 1991) (Gieselmann et al., 1989) (Regis et al., 1998)
MLD2	FFF0711993/ Gaslini Biobank	fetal	[459+1G>A] + [459+1G>A]	[c.465+1G>A] + [c.465+1G>A]	Splicing site in intron 2 + Splicing site in intron 2	LI	(Berger et al., 1999) (Polten et al., 1991)
ND1	FFF0561980/ Gaslini Biobank	adult					
ND2	C0045C/ Invitrogen	newborn					

Table B

Clone ID	Donor ID	Therapeutic Vector	ARSA activity (nmol/h*mg)
MLD-ARSA 1.1	MLD 1	bd.LV.hARSA.GFP	2,407.9 ± 774.7
MLD-ARSA 1.2		bd.LV.hARSA.GFP	2,340.3 ± 1,836.5
MLD-ARSA 1.3		LV.hARSA	4,915.1 ± 1,229.0
MLD-ARSA 2.1	MLD 2	LV.hARSA	10,918.4 ± 2,192.5
MLD 1.1	MLD 1	N.A.	71.6 ± 9.4
MLD 1.2		N.A.	63.5 ± 16.3
MLD 1.3		N.A.	54.9 ± 14.1
MLD 2.1	MLD 2	N.A.	33.3 ± 10.1
ND 1.1	ND 1	N.A.	633.5 ± 90.4
ND 1.3		N.A.	920.2 ± 130.6
ND 2.2	ND 2	N.A.	369.2 ± 66.3
ND 2.3		N.A.	652.7 ± 101.8

Cell culture

iPSCs were maintained on mitomycin-C-treated MEF in iPSCM and split using collagenase IV (Gibco) at a ratio from 1:3 to 1:6. For neural differentiation, iPSCs were plated on Matrigel-coated dishes in MEF-conditioned IPSCM. The medium was then changed daily according to the protocol described in Results. The composition of culture media is provided in the table below.

Cell culture media	Components	Provider	Concentration
iPSCM	**DMEM-F12**	Life Technologies	
	Knockout serum replacement	Life Technologies	20%
	Sodium Bicarbonate 7.5%	Life Technologies	0.18%
	Non-essential Aminoacids	Life Technologies	0.1 mM
	Penicillin-streptomycin	Lonza	100 U/ml
	L-glutamine	Lonza	2 mM
	Sodium pyruvate	Life Technologies	1 mM
	β-mercaptoethanol	Life Technologies	0.1 mM
	FGF2	Peprotech	10 ng/ml
KSRM	**KO DMEM**	Life Technologies	
	Knockout serum replacement	Life Technologies	15%
	Non-essential Aminoacids	Life Technologies	0.1 mM

	Penicillin-streptomycin	Lonza	100 U/ml
	L-glutamine	Lonza	2 mM
	β-mercaptoethanol	Life Technologies	0.1 mM
**NPCM**	**DMEM F12**	Life Technologies	
	D-glucose	Sigma	9 mM
	Sodium bicarbonate	Sigma	23.8 mM
	Putrescin	Sigma	16 mg/l
	Progesteron	Sigma	6.4 μg/l
	Sodium Selenite	Sigma	30 nM
	Transferrin	Sigma	0.1 g/l
	Insulin	Sigma	0.22 μM
	Non-essential Aminoacids	Life Technologies	0.1 mM
	Penicillin-streptomycin	Lonza	100 U/ml
	Glutamax	Life Technologies	1X
**GDM**	**NPCM**		
	B27 supplement	Life Technologies	1X
	triiodothyronine (T3)	Sigma	60 ng/ml
	Biotin	Sigma	100 ng/ml
	dibutyryl cyclic AMP	Sigma	1 μM
	Insulin	Sigma	25 μg/ml
	Platelet derived growth factor alpha (PDGF AA)	Peprotech	10 ng/ml
	Insulin growth factor-1 (IGF1)	Peprotech	10 ng/ml
	Neurotrophin-3 (NT3)	Peprotech	10 ng/ml
	Hepatocyte growth factor (HGF)	Peprotech	5 ng/ml
**GMM**	**NPCM**		
	B27 supplement	Life Technologies	1X
	triiodothyronine (T3)	Sigma	60 ng/ml
	Biotin	Sigma	100 ng/ml
	dibutyryl cyclic AMP	Sigma	1 μM
	Insulin	Sigma	25 μg/ml

Lentiviral-mediated gene transfer

iPSC colonies were manually picked, replated on Matrigel-coated dishes, and expanded for one passage in iPSC medium supplemented with 10 μM Y-27632 (Sigma). After 3 days, iPSCs were dissociated at single cells with Accutase and plated as single cells on Matrigel-coated 12 well dishes (20,000 cells/well) in iPSC medium supplemented with 10 μM Y-27632. The day after, cells were

transduced with bdLV.GFP.hARSA-HA or with LV.hARSA at MOI 100 in iPSC medium supplemented with 10 μM Y-27632 and 8 μg/ml Polybrene, as previously described (Meneghini et al., 2017). After 24 hours, the medium was replaced with fresh iPSC medium supplemented with 10 μM Y-27632. When confluence was reached, cells were detached with Dispase (Life Technologies) and plated on mitomycin C-inactivated MEFs. iPSC medium was replaced daily till the formation of iPSC colonies.

Human brain tissues

Post-mortem snap-frozen brain samples (periventricular white matter, PWM; cerebral cortex, Ctx) from 4 MLD patients and 4 age-matched normal donors (ND) were obtained from the University of Maryland Brain and Tissue Bank (a Brain and Tissue Bank repository of the NIH NeuroBioBank; https://neurobiobank.nih.gov) under a specific MTA (request ID: 616). ARSA mutations have been determined as previously described (Cesani et al., 2016) and are reported according to current nomenclature guidelines ascribing the A of the first ATG translational initiation codon as nucleotide +1 (http://www.hgvs.org/mutnomen). ARSA activity has been measured using the artificial substrate MUS (see also Supplementary Figure S3); nd, not detected; na, not available.

Donor ID		Sex/ age (years)	Mutation	Pseudo-deficiency	Brain region	ARSA activity (nmol*mg/h)
MLD	#5745	Female/3	c.641C>T c.1010A>T	nd	PWM	nd
					Ctx	
	#5505	Male/3	c.847G>T c.1010A>T	N350S *96A>G	PWM	
					Ctx	
	#5785	Female/17	na	na	PWM	
					Ctx	
	#M3308	Female/21	c.465+1G>A c.585G>C	nd	PWM	
					Ctx	
ND	#4369	Female/2	nd		PWM	6.38
					Ctx	1.87
	#5941	Male/2			PWM	6.69
					Ctx	2.09
	#1038	Female/24			PWM	5.71
					Ctx	2.93
	#4541	Female/22			PWM	5.34
					Ctx	2.67

Gene expression studies

mRNA was extracted from cell pellets (1-2x10^6 cells) with RNeasy mini Kit (Qiagen) according to the manufacturer instructions. Quantification of mRNA was determined by 260/280 nm OD reading on the NanoDrop ND-1000 spectrophotometer. Reverse transcriptase reactions were performed using

QuantiTect reverse transcription kit (Qiagen) according to the manufacturer instructions. RT-PCR analysis were performed by using FastStartTM Taq DNA Polymerase, PCR KIT (Roche). For each reaction, 100 ng of template cDNA and 1 mM of primers (listed below) were used. PCR products were then resolved by electrophoresis in a 1.5% agarose gel. Taqman analyses were performed in Optical 96-well Fast Thermal Cycling Plates on Viia7 Real-Time PCR System by using Universal PCR Master Mix. For each reaction, 100 ng of template cDNA and commercial TaqMan Gene Expression Assays (listed below) (Applied Biosystems) were used.

**RT-PCR**	
**Gene**	**Primer Sequence**
SOX2	For 5'-TTACCTCTTCCTCCCACTCCAG-3'
	Rev 5'-GGGTTTTCTCCATGCTGTTTCT-3'
NANOG	For 5'- GAT CGG GCC CGC CAC CAT GAG TGT GGA TCC AGC TTG-3'
	Rev 5'- GAT CGA GCT CCA TCT TCA CAC GTC TTC AGG TTG-3'
GAPDH	For 5'- CAG CCT CAA GAT CAT CAG CA -3'
	Rev 5'- TGC TGT AGC CAA ATT CGT TG -3'
PAX6	For 5'- AAC AGA CAC AGC CTT CAC AAA CA-3'
	Rev 5'- CGG GAA CTT GAA CTG GAA CTG AC-3'
**TaqMan**	
**Gene**	**Probe code**
hPAX6	Hs00240871_m1
hGAPDH	Hs02758991_g1
hHPRT1	Hs01003267_m1
hOCT4	Hs03005111_g1
hGAL3ST	Hs00191582_m1
hCGT	Hs00409961_m1
hGALC	Hs01012300_m1
hARSA	Hs04185629_g1

Immunofluorescence

Cells fixed in 4% Paraformaldehyde (PFA) were rinsed with PBS, incubated with blocking solution containing 10% normal goat serum (NGS) + 0.1% Triton X-100 in PBS for 30 minutes and left o/n at 4°C with primary antibodies (listed below) diluted in blocking solution. After 3 washes (5 minutes each), antibody staining was revealed using species-specific fluorophore-conjugated secondary antibodies (listed below) diluted in 1% NGS in PBS for 1 hour at room temperature. Finally, cells were

counterstained with ToPro-3 (Invitrogen) or 4', 6-diamidino-2-phenylindole (DAPI, Roche) for detection of nuclei. Coverslips were mounted on glass slides using Fluorsave (Calbiochem). Samples incubated only with secondary antibodies were used as negative controls.

Primary antibodies		
**Antigen**	**Host species (provider, product number)**	**Dilution**
β-tubulin III	Rabbit polyclonal (Babco, PRB-435P)	1:1,000
GFAP	Mouse monoclonal (Millipore, MAB3402)	1:2,000
NESTIN	Mouse monoclonal (Millipore, MAB353)	1:200
Antigen Ki67 (Ki67)	Mouse monoclonal (Novocastra, NCL-Ki67-MM1)	1:100
Cleaved caspase 3	Rabbit polyclonal (Cell Signaling 9661)	1:200
PAX6	Rabbit polyclonal (Biolegend PRB-278P)	1:200
A2B5	Mouse monoclonal (Chemicon MAB312)	1:1,000
NG2	Rabbit polyclonal (Chemicon MAB5320)	1:300
OLIG2	Rabbit polyclonal (Abcam AB136253)	1:300
APC	Mouse monoclonal (Calbiochem OP80)	1:500
CNPase	Mouse monoclonal (Chemicon MAB326R)<br>Rabbit monoclonal (Cell Signaling D83E10)	1:500<br>1:150
MBP	Rat monoclonal (Chemicon MAB386)	1:300
FOXG1	Rabbit polyclonal (Abcam AB18259)	1:300
NF200	Mouse monoclonal (Sigma Aldrich N 0142)	1:300
Sulfatide	Mouse monoclonal<br>(kindly provided by M. Blomqvist, Gothenburg, Sweden)	1:100
LAMP1	Rabbit polyclonal (Abcam AB24170)	1:500
**Secondary antibodies**		
**Fluorochrome**	**Host and target (provider, product number)**	**Dilution**
Alexa 488	Goat anti-mouse IgG (Mol.Probes, A11001)<br>Goat anti-rabbit IgG (Mol.Probes, A11008)<br>Goat anti-rat IgG (Mol.Probes, A11006)	1:1,000
Alexa 546	Goat anti-mouse IgG (Mol.Probes, A11003)<br>Goat anti-rabbit IgG (Mol.Probes, A11010)	1:2,000

ROS analysis

For ROS determination, iPSCs and differentiated cells were incubated with CellROX® Deep Red Reagent (Thermo Scientific) diluted 1:500 in their correspondent media for 15 minutes at 37° C. After

2 washes in PBS, nuclei were counterstained with Hoechst. Cells were then rinsed in Krebs-Ringers Henseleit (KRH) and mounted.

Lysotracker analyses

Determination of lysosomes number was performed using a high content imaging platform in the Advanced Light and Electron Microscopy Bio-Imaging Centre of San Raffaele Scientific Institute (ALEMBIC) using the ArrayScan XTI HCA Reader from ThermoFisher Scientific.

Cells were plated on matrigel-coated 96 Greiner Sensoplate glass bottom multiwell plates (Sigma Aldrich) at a density of 6.000 cells/well. For lysosomal marking, cells are incubated with 50 nM LysoTracker Red DND-99 (Thermo Fisher) and 0.5 μM CellTracker Green CMFDA (Thermo Fisher) for cell identification, diluted in culture medium, for 20 minutes at 37°C. Nuclear staining was performed by Hoechst (5 min at room temperature, 10 μg/ml final concentration). Cells are then washed with Krebs-Ringer-Hepes-bicarbonate buffer (KRH) and maintained in the same buffer for the entire duration of the instrument acquisition. Fifty fields were acquired for each well; images were analysed with the Thermo Scientific HCS Studio Cell Analysis Software.

Electromicroscopy analyses

iPSCs were fixed for 15 min at 4 °C with 4% PFA and 2.5% glutaraldehyde in 125 mM cacodylate buffer, scraped and then centrifuged at 13.000 x g. The pellet was post-fixed (1 hour) with 2% OsO_4 in 125 mM cacodylate buffer, washed, dehydrated and embedded in Epon (Zuka). Conventional thin sections were collected on uncoated grids, stained with uranyl and lead citrate and examined in a Leo912 electron microscope (ALEMBIC). Lysosomes were identified as electron dense rounded vesicles with lipid droplets. Lysosomal area (expressed in pixels) was calculated using the ImageJ software. Results were expressed as a percentage of ND values. At least 10 fields/sample were quantified in n=3 experiments performed in triplicates.

Western blot

Cell pellets (2-3 x 10^6 cells) were resuspended in 30μl of RIPA buffer (50 mM TRIS HCl pH 7.4-7.5, 150 mM NaCl, 0.5% Deoxycholate (DOC), 0.1% Sodium Dodecyl Sulphate (SDS), 2mM EDTA, 1% Triton) supplemented with protease inhibitor (EDTA-Free Protease Inhibitor Cocktail, Roche) and phosphatase inhibitor 10X (PhosphoSTOP, Roche). The cell suspension was subjected to 3 rounds of sonication (three cycles of 15 pulses, 0.5 seconds/pulse, 0.8 amplitude). We subsequently operated 3 freeze/thaw cycles of 3' each in 37°C bath and dry ice. Lysates were centrifuged at 12.000 x *g* for 10' at 4°C, and supernatants were used as protein extracts for biochemical analysis. We measured

protein content using the Bradford Protein Assay kit with bovine serum albumin (BSA) as the reference standard. 10-20 μg of samples were prepared and loaded on 4-12% Novex NuPAGE SDS-PAGE system according to manufacturer instructions. The PVDF membranes were stained with the antibody listed in below, revealed by HRP-conjugated goat anti-rabbit (1:10,000; AP132P, Chemicon), chicken anti-goat (1:10,000; sc2153, Santa Cruz), goat anti-mouse (1:10,000; AP124P, Chemicon) and incubated with chemiluminescent substrate (EMD Millipore Immobilion Western Chemiluminescent HRP Substrate).

**Primary antibodies for WB analysis**		
**Antigen**	**Host species (company, catalog number)**	**Dilution**
PDGFRα	Rabbit polyclonal (Santa Cruz sc338)	1:200
CNPase	Mouse monoclonal (Millipore MAB326R)	1:1,000
GFAP	Rabbit polyclonal (Dako ZO334)	1:10,000
β-tubulin III	Mouse monoclonal (Millipore MAB	1:5,000
LAMP1	Rabbit polyclonal (Abcam AB24170)	1:2,000
EEA1	Rabbit polyclonal (ABR PA1-063)	1:2,000
GM130	Mouse monoclonal (BD Pharmigen 610823)	1:600
β-actin	Goat polyclonal (Santa Cruz sc-1616)	1:5,000
Synaptotagmin 1	Mouse monoclonal (Synaptic Systems 105 011)	1:10,000

Analysis of sulfatide content

Pellets of cells were transferred to 2 ml polypropylene tubes and 6 zirconium oxide beads (3 mm) were added. The internal standard (C19:0 sulfatide), dissolved in 500 μl butanol:methanol (3:1, v/v), were then added and the samples were homogenized using a Mixer Mill 301 instrument (Retsch GmbH, Haan, Germany). Lipid extraction was continued by adding 500 μl heptane:ethyl acetate (3:1) and 500 μl acetic acid (1% in water). After another 5 minutes of homogenization and mixing using the Mixer Mill 301 instrument the samples were centrifuged at 20 000 g for 5 minutes. The upper layer was removed using a Pasteur pipette and evaporated. Finally, the samples were dissolved in chloroform:methanol:water (3:6:2; v/v/v) for analysis.

Sulfatide species were quantified using ultra-performance liquid chromatography-tandem mass spectrometry (UPLC-MS/MS). For the separation of sulfatide species, a Kinetex C18 column was used (2.1x100 mm 1.7 μm; Phenomenex, Torrance, CA, USA). The mobile phases consisted of A) water: acetonitrile: formic acid (30:70:0.1) and B) acetonitrile: isopropanol: formic acid (50:50:0.1). Detection and quantification of the sulfatides were made using a QTRAP 5500 mass spectrometer (Sciex,

Concord, Canada). The analysis was made in negative mode using multiple reaction monitoring (MRM) with the sulfate ion (m/z 96.8) as fragment ion.

Quantification of ARSA activity

Cells. Cell pellets (2-3 x 10^6 cells) were resuspended in 50 µl of lysis buffer (Sodium acetate 0,05 M) and then subjected to 3 rounds of sonication (three cycles of 15 pulses, 0.5 seconds/pulse, 0.8 amplitude). We subsequently performed 5 freeze/thaw cycles in 37°C bath and dry ice. Then lysates were centrifuged at 16,000 x g for 10 minutes at 4 °C, and supernatants were used as protein extracts for biochemical analysis. We measured protein content using the Bradford Protein Assay kit with BSA as the reference standard. We prepared samples of 300 ng/µl diluted in sodium acetate 0.05 M to a final volume of 20 µl, then we added 20 µl of *p*-nitrocathecol solution (1:1) and incubated samples for 1 hour at 37°C. The *p*-nitrocatechol reaction was stopped with 200 µl of NaOH 1 M. Absorbance of the samples was read at ELISA reader at λ=515 nm as optical density (OD). ARSA activity was calculated as follows:

- nmol/ml = (OD/0.0105)*25 where 0,0105 is the extinction coefficient of *p*-nitrocathecol at 515 nm and 25 is the normalization for 40 µL of solution.
- nmol/mg = (nmol/ml) / (µg/µl proteins loaded)
- nmol/mg*h (correction for time of incubation)

Human brain tissues. ARSA activity in frozen tissues was determined using the 4-methylumbelliferyl-sulfate substrate (MUS) dissolved in 0.05 M Na-acetate/acetic acid buffer, pH 5.5, in the presence or absence of 125 mM $AgNO_3$ (a specific ARSA inhibitor) at 378C (90). ARSA activity was calculated by subtracting the value obtained in the presence of $AgNO_3$ (arylsulfatase B – ARSB activity) from that measured in the absence of the inhibitor (ARSA + ARSB activity). All enzymatic reactions were stopped by adding 0.2M glycine/NaOH, pH 10.6. Fluorescence of liberated 4-methylumbelliferone was measured on a spectrofluorometer (λ_{ex} 360 nm; λ_{em} 446 nm)(Lattanzi et al., 2010; Martino et al., 2005)

Microelectrode Array (MEA) experiments and analysis.

iPSC-NPCs were plated for differentiation on sterile MEAs chip pre-treated 1 hour with Matrigel. Electrophysiological extracellular recording of iPSC-derived neurons was performed at 14 and 24 days in vitro with standard 60-electrode MEA chips with 30 µm electrode diameter, 200 µm inter-electrode spacing (Multichannel Systems GmbH, MCS GmbH, Reutlingen, Germany). The setup for MEA recordings was composed of a pre-amplifier stage (MEA1060- Inv-BC-Standard, gain: 55, bandwidth: 0.02Hz–8.5kHz, MCS GmbH), an amplification and filtering stage (FA64S, gain: 20, bandwidth: 10Hz–

3 kHz, MCS GmbH) and a data acquisition device (USB-ME64, sampling frequency: 25 kHz, MCS GmbH). Experiments have been performed at 37°. Recordings were carried out under control condition and in presence of the voltage-dependent sodium channel blocker tetrodotoxin (TTX) (Tocris, Bristol, United Kingdom). The spontaneous electrophysiological activity was monitored for 5minutes at the beginning of each experiment to reach a stable level of the electrical signal and then for 5minutes for the spike recording. We analyzed MEA data using an off-line spike detection with MC_Rack Software (MCS GmbH) appointing a channel-specific threshold equal to 5-folds the standard deviation of the average noise amplitude during the first 500ms of recording. The subsequent spike analysis was implemented in Matlab (The Mathworks, Natick, USA), as previously described (Rubio et al., 2016).

Supplemental references

Berger, J., Gmach, M., Mayr, U., Molzer, B., and Bernheimer, H. (1999). Coincidence of two novel arylsulfatase A alleles and mutation 459+1G>A within a family with metachromatic leukodystrophy: Molecular basis of phenotypic heterogeneity. Hum. Mutat. *13*, 61–68.

Blomqvist, M., Borén, J., Zetterberg, H., Blennow, K., Månsson, J.-E., and Ståhlman, M. (2017). High-throughput analysis of sulfatides in cerebrospinal fluid using automated extraction and UPLC-MS/MS. J. Lipid Res. *58*.

Cesani, M., Lorioli, L., Grossi, S., Amico, G., Fumagalli, F., Spiga, I., Filocamo, M., and Biffi, A. (2016). Mutation Update of ARSA and PSAP Genes Causing Metachromatic Leukodystrophy. Hum. Mutat. *37*, 16–27.

Gieselmann, V., Polten, a, Kreysing, J., and von Figura, K. (1989). Arylsulfatase A pseudodeficiency: loss of a polyadenylylation signal and N-glycosylation site. Proc. Natl. Acad. Sci. U. S. A. *86*, 9436–9440.

Lattanzi, A., Neri, M., Maderna, C., di Girolamo, I., Martino, S., Orlacchio, A., Amendola, M., Naldini, L., and Gritti, A. (2010). Widespread enzymatic correction of CNS tissues by a single intracerebral injection of therapeutic lentiviral vector in leukodystrophy mouse models. Hum. Mol. Genet. *19*, 2208–2227.

Martino, S., Consiglio, A., Cavalieri, C., Tiribuzi, R., Costanzi, E., Severini, G.M., Emiliani, C., Bordignon, C., and Orlacchio, A. (2005). Expression and purification of a human, soluble Arylsulfatase A for Metachromatic Leukodystrophy enzyme replacement therapy. J Biotechnol *117*, 243–251.

Meneghini, V., Frati, G., Sala, D., De Cicco, S., Luciani, M., Cavazzin, C., Paulis, M., Mentzen, W., Morena, F., Giannelli, S., et al. (2016). Generation of Human Induced Pluripotent Stem Cell-Derived Bona Fide Neural Stem Cells for Ex Vivo Gene Therapy of Metachromatic Leukodystrophy. Stem Cells Transl. Med. 1–17.

Nelson, P. V., Carey, W.F., and Morris, C.P. (1991). Population frequency of the arylsulphatase A pseudo-deficiency allele. Hum. Genet. *87*, 87–88.

Polten, A., Fluharty, A.L., Fluharty, C.B., Kappler, J., von Figura, K., and Gieselmann, V. (1991). Molecular basis of different forms of metachromatic leukodystrophy. N Engl J Med *324*, 18–22.

Regis, S., Filocamo, M., Stroppiano, M., Corsolini, F., Caroli, F., and Gatti, R. (1998). A 9-bp deletion (2320del9) on the background of the arylsulfatase A pseudodeficiency allele in a metachromatic leukodystrophy patient and in a patient with nonprogressive neurological symptoms. Hum. Genet. *102*, 50–53.

Rubio, A., Luoni, M., Giannelli, S.G., Radice, I., Iannielli, A., Cancellieri, C., Di Berardino, C., Regalia, G., Lazzari, G., Menegon, A., et al. (2016). Rapid and efficient CRISPR/Cas9 gene inactivation in human neurons during human pluripotent stem cell differentiation and direct reprogramming. Sci. Rep. *6*.
